# Microcellular Injection-Molded LDPE/Activated Carbon Composites: Material Properties and Arsenic [As(III)/As(V)] Adsorption Performance

**DOI:** 10.3390/polym18212656

**Published:** 2026-10-26

**Authors:** Minyuan Chien, Naveen Bunekar, Tlou Nathaniel Moja, Shyhshin Hwang, Peter Hsu

**Affiliations:** 1 Department of Mechanical Engineering, Chien-Hsin University of Science and Technology, Taoyuan 320678, Taiwan; jackyaren@uch.edu.tw; 2 Department of Chemistry, Chung Yuan Christian University, Taoyuan 320314, Taiwan; naveen.bunekar@gmail.com; 3 Institute for Nanotechnology and Water Sustainability (iNanoWS), College of Science, Engineering and Technology, University of South Africa, Florida Campus, Johannesburg 1709, South Africa; 4 R & D, Kemflo International Co., Ltd., Pingtung 900509, Taiwan; peter.hsu@kemflointernational.com.tw

**Keywords:** microcellular injection molding, low-density polyethylene (LDPE), activated carbon, polymer composite foam, supercritical nitrogen (scN_2_), arsenic adsorption, As(III)/As(V) remediation, water treatment

## Abstract

Arsenic contamination in water bodies caused by acid mine drainage (AMD) and industrial effluents presents severe threats to public health and aquatic ecosystems. Although activated carbon (AC) is an effective adsorbent, conventional powdered AC suffers from severe operational limits, including filter clogging, high pressure drop, and secondary pollution. Encapsulating AC inside dense low-density polyethylene (LDPE) matrices resolves powder handling issues but severely restricts contaminant mass transport. In this study, novel lightweight LDPE/AC composite foams (1, 3, 6, and 10 wt% AC) were fabricated using microcellular injection molding with supercritical nitrogen (scN_2_). The microcellular architecture thinned the polymer cell walls and generated an interconnected porous cell network, exposing embedded AC particles directly to the aqueous media. Morphological and thermal analyses confirmed uniform filler dispersion and preserved matrix stability, with the 10 wt% AC composite foam reaching a high BET surface area of 374 m^2^/g. Tensile modulus increased with AC loading up to 2535 MPa. Batch adsorption studies demonstrated optimal removal efficiency for As(III) at pH 7–8 and As(V) at pH 3, achieving maximum adsorption capacities of 20.71 mg/g and 24.00 mg/g, respectively. Treated water streams successfully met the WHO and South African SANS 241:2015 drinking water standard of 10 ppb. The adsorption dynamics obeyed pseudo-second-order kinetics and fitted the Freundlich isotherm model, indicating multi-layer chemisorption across heterogeneous surfaces. Desorption using 0.1 M EDTA retained high efficiency over 10 consecutive cycles. This work demonstrates microcellular LDPE/AC composite foams as a scalable, high-strength, and durable material platform for heavy metal remediation.

## 1. Introduction

Polymer composites play a vital role in modern materials engineering due to their ability to combine matrix processability with filler functionality. Fillers serve diverse roles in polymer systems, including enhancing mechanical performance, improving thermal properties, providing UV resistance, and reducing overall production costs [1]. However, the compatibility between the filler and the polymer matrix remains a fundamental challenge. Because thermoplastic matrices are generally hydrophobic whereas most functional fillers are hydrophilic, surface modification or specific physical integration strategies are frequently required to achieve strong interfacial adhesion. Low-density polyethylene (LDPE) is widely preferred as a matrix resin or carrier polymer for functional fillers due to its unique structural and processing advantages. LDPE possesses a highly branched molecular structure featuring long-chain branching [2]. Under high-shear processing—such as injection molding—these branched chains realign easily, inducing pronounced shear-thinning behavior. This rheological characteristic enables highly filled formulations to flow smoothly through dies and mold cavities without generating extreme backpressure or equipment strain.

Beyond structural applications, engineering functional polymer composites for environmental remediation has gained significant momentum. Groundwater contamination by heavy metals represents a major global environmental crisis [3]. Arsenic (As), ranked among the world’s top 20 hazardous substances, exists in both organic and inorganic forms. In aquatic environments, arsenic occurs predominantly as inorganic species across different redox states: arsenite [As(III)] and arsenate [As(V)] [4]. Conversely, organic species are typically introduced via anthropogenic activities, riverbed erosion, and sediment deposition [5]. The toxicity of arsenic varies significantly depending on the chemical species and biological detoxification mechanisms. Generally, inorganic arsenic is far more toxic than organic forms and is classified as a Group 1 human carcinogen [6]. Specifically, As(III) exhibits higher acute toxicity than As(V), while methylated intermediates such as monomethylarsonous acid (MMA^III^) and dimethylarsinous acid (DMA^III^) display even higher toxicity than their parent compounds [7].

To mitigate arsenic exposure, regulatory bodies—including the World Health Organization (WHO), the United States Environmental Protection Agency (US-EPA), and South African National Standards (SANS 241-1:2015)—have established a strict maximum contaminant level of 10 parts per billion (ppb), or 0.010 mg/L, in drinking water [8]. Globally, inorganic arsenic exposure affects more than 200 million people, leading to severe chronic health conditions including nervous system dysfunction, kidney failure, liver disease, cardiovascular disorders, and cancers of the skin, lungs, and urinary bladder. In South Africa, extensive arsenic contamination of groundwater has been documented in the Mpumalanga mining region, particularly around eMalahleni [9]. Acid mine drainage (AMD) from abandoned mining operations poses a severe threat as runoff contaminates local soils and water bodies. Conventional heavy metal remediation methods—such as chemical precipitation [10,11,12], ion exchange [13], crystallization, membrane filtration [14], lime neutralization [15], and biological wetlands [16]—are often costly due to high energy consumption, heavy reagent requirements, and complex sludge disposal. Moreover, conventional techniques often suffer from low efficiency in treating low-concentration streams (<1 mg/L) and display slow operating kinetics [17].

Adsorption utilizing low-cost polymer-supported adsorbents offers a highly sustainable alternative [18]. Various low-cost carbonaceous and inorganic precursors—including concrete aggregates [19], granular activated carbon [20], red mud/steel slag [21], coal fly ash [22,23,24], sawdust [25], clay minerals [26], agro-industrial waste [27], and chitosan [28,29,30]—have been explored. Among these, activated carbon (AC) is a premier adsorbent due to its high specific surface area, hierarchical porous structure, and rich surface functional groups (COOH, -OH, and C=O). For example, Kumar et al. (2023) [31] demonstrated that charcoal-derived AC from *Zea mays* (surface area increased from 29.85 m^2^/g to 422 m^2^/g post-activation) removed As(III) within 60 min. However, that study did not evaluate adsorption kinetics, equilibrium models, or regeneration performance—metrics essential for practical fixed-bed system design. Furthermore, fine powdered activated carbon (PAC) poses severe practical challenges: it causes filter clogging, high pressure drops, dusting, and difficult post-treatment solid–liquid separation, which can lead to secondary pollution.

To overcome the fluid-handling limitations of unbound powders, the previous literature has extensively investigated embedding activated carbon and related carbonaceous fillers into non-porous LDPE matrices [32,33,34,35]. Early studies on solid LDPE/AC composites focused primarily on evaluating thermal stability, electrical conductivity, and mechanical reinforcement resulting from three primary interfacial interaction mechanisms: native interactions, covalently modified interactions, and mechanical interlocking [35,36]. Subsequent research extended LDPE/AC composites to environmental applications, demonstrating that immobilizing AC within an LDPE matrix successfully eliminates powder dusting and secondary waste disposal [37]. However, these conventional solid LDPE/AC composite adsorbents revealed a critical performance bottleneck: because LDPE forms a dense, non-porous hydrophobic encapsulation, the majority of embedded AC particles remain deeply buried inside the bulk polymer matrix [38]. As a result, aqueous metal species face severe intraparticle diffusion resistance, preventing them from contacting active binding sites. Consequently, solid LDPE/AC composites exhibit sluggish adsorption kinetics, low total capacity, and incomplete active site utilization compared to unbound AC powders [35].

Microcellular injection molding (e.g., the MuCell process) offers a compelling strategy to resolve this encapsulation trade-off. This process utilizes nitrogen (N_2_) as a physical blowing agent, which is compressed above 330 bar into a supercritical state (scN_2_) and injected into the extruder barrel to mix with the polymer melt, forming a homogeneous single-phase solution under high pressure (~100 bar). Upon injection into the mold cavity, rapid pressure drop induces thermodynamic instability, triggering cell nucleation and subsequent bubble growth. The incorporation of functional fillers within the polymer matrix provides additional heterogeneous interfacial nucleation sites, yielding smaller, more uniform microcellular pore structures [39]. Furthermore, the long-chain branching architecture of LDPE profoundly enhances its foaming capability [40], providing melt strength during bubble growth. Crucially, microcellular foaming thins out the polymer cell walls and creates an interconnected micro-foamed network. This porous architecture exposes a vastly higher percentage of embedded AC binding sites directly to fluids, drastically shortening intraparticle diffusion pathways and enabling rapid adsorption kinetics.

Despite these clear advantages, research on physical microcellular foamed LDPE/AC composites for heavy metal adsorption—specifically comparing the simultaneous removal mechanisms of As(III) and As(V)—remains extremely scarce. Therefore, this study aims to synthesize novel, low-cost microcellular LDPE/AC composite foams using supercritical N_2_ injection molding. The objectives of this work are to fabricate microcellular LDPE/AC composites across various AC loading fractions and systematically evaluate their morphological, thermal, and mechanical properties. Investigate the simultaneous immobilization and adsorptive removal performance toward both toxic As(III) and As(V) species under varying operational parameters (pH, adsorbent dosage, contact time, and initial concentrations). Evaluate whether the treated water streams can meet the stringent SANS 241:2015 drinking water threshold (10 ppb) [41]. Elucidate the underlying adsorption kinetics, isotherm behavior, regenerability, and structural mass-transport pathways governed by the microcellular foam architecture.

## 2. Materials and Methods

### 2.1. Materials

Raw black activated carbon (AC) powder of mesh number (200 × 400) with a particle size of 1214 nm was supplied by Kemflo International Co., Ltd., Pingtung, Taiwan. LDPE resin pellets (3 mm in particle size), grade NA 248 with a melt flow index of 48 g/10 min, were procured from USI Corporation, Kaohsiung, Taiwan; both materials were used as the adsorbents. Sigma-Aldrich Pty Ltd., Jet Park, South Africa, supplied a multi-element standard solution (40 mg/L of As) for ICP. This solution was diluted with deionized water with a resistivity of 18 MΩ. Analytical-grade sodium arsenate dibasic heptahydrate (Na_2_HAsO_4_·7H_2_0) and sodium arsenite (NaAsO_2_), purchased from Sigma-Aldrich Pty Ltd., were dissolved in deionized water to obtain As(III) and As(V) stock solutions (20 mg/L). All plasticware and glassware were soaked in 2% (*v*/*v*) HNO_3_, followed by repeated rinsing with deionized water, and dried before use. The AC content was varied at 1, 3, 6, and 10 wt%.

### 2.2. Methods

#### 2.2.1. Foamed Injection Molding Machine and Mold

**Figure 1:**
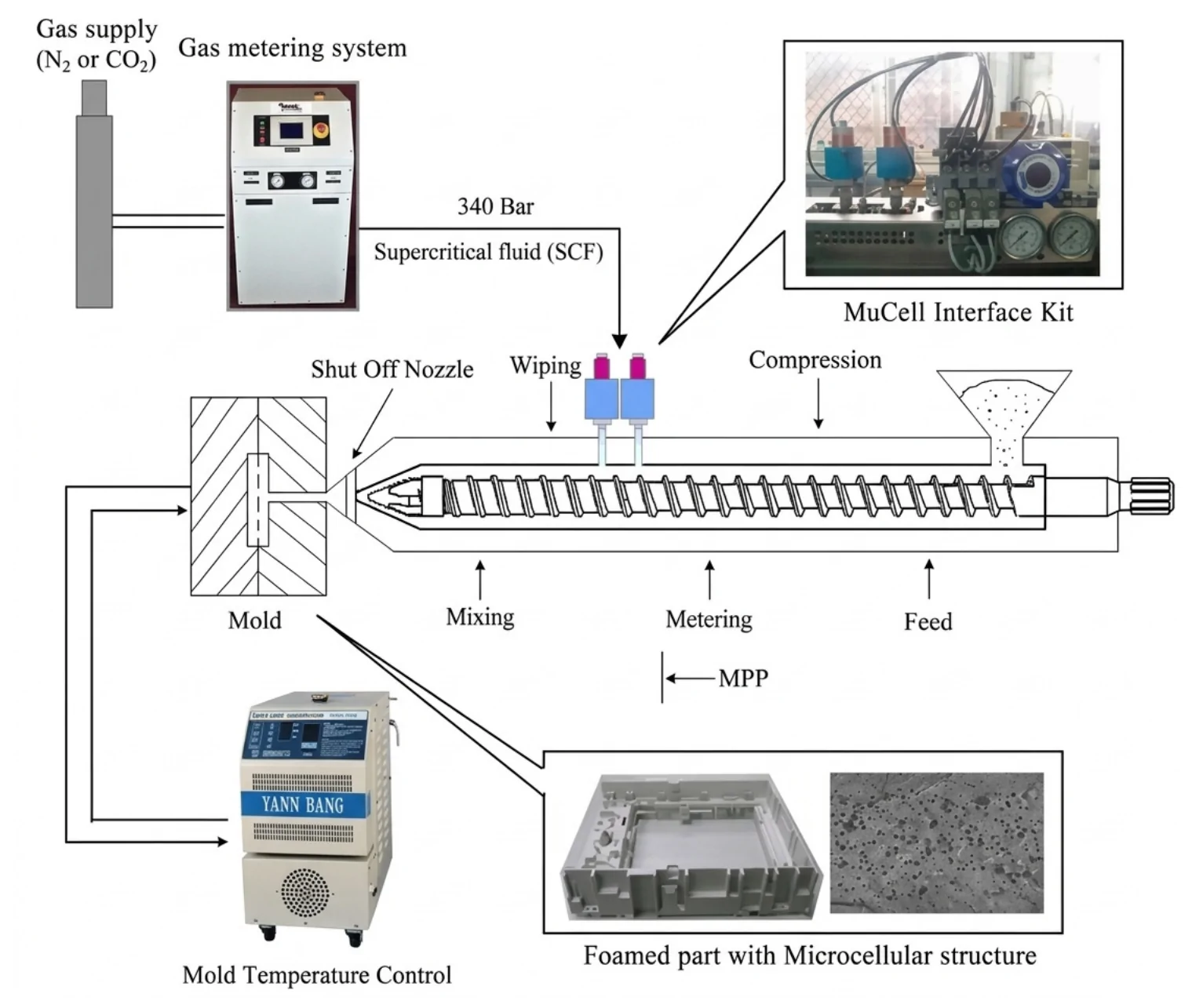
Microcellular injection molding setup.

Composites of AC and LDPE were prepared by coating the surface of LDPE pellets with pre-mixed black AC powder. The mixture was then fed into the hopper of an injection molding machine. Microcellular injection molding was performed using a 100-ton Arburg 420C (Lossberg, Germany) injection molding machine equipped with MuCell^®^ (Trexel, Boston, MA, USA) capability. Nitrogen served as the physical blowing agent. A supercritical fluid (SCF) booster pumped nitrogen gas up to 34 MPa and injected the SCF into the polymer melt on the mixing section of the screw. A shutoff nozzle kept the polymer melt as a single phase at around 10 MPa in the barrel. Upon injection, thermal instability occurred, causing cell nucleation and cell growth. Consequently, a foamed polymer was formed (Figure 1). The mold utilized was an ASTM D638 type I tensile bar mold. The foamed specimens were then tested for their mechanical properties and adsorption capacities. Table 1 presents the process parameters used for foamed injection molding.

#### 2.2.2. Adsorption Experiments

The adsorption kinetics and isotherms for As(III) and As(V) adsorption onto LDPE and AC were measured in triplicate. Stock solutions of 100 mg/L As(III) and As(V) were diluted with deionized water or river water to obtain a range of initial As(III) and As(V) solutions (0.5, 1.0, 2.0, 4.0, 8.0, and 10 mg/L). For the kinetic study, a mixture of approximately 0.5 g of LDPE/AC and 250 mL of the diluted arsenic solution (10.0 mg/L) was placed in a 500 mL beaker and agitated on a horizontal shaker at 150 rpm for 5 to 50 min. In addition, the adsorption of As(III) and As(V) onto river sediment was conducted by mixing 0.5 g of LDPE/AC and 2.0 g of river sediment in 200 mL of a 10.0 mg/L As(III)/As(V) solution prepared in deionized water or river water for 7 days. During the adsorption process, 3 mL suspension samples were taken at regular intervals (5, 10, 15, 20, 25, 30, 35, 40, 45, and 50 min), filtered through a 0.45-μm membrane filter, and analyzed for total As using ICP-OES (Agilent, Santa Clara, CA, USA). The adsorption of As(III) and As(V) was evaluated by monitoring the decrease in arsenic concentration in the solution.

### 2.3. Kinetic and Equilibrium Sorption Studies

The study utilized a systematic batch adsorption methodology to evaluate the effects of pH, initial metal concentration, and contact time on the removal of As(III) and As(V). For each experiment, approximately 2.0 g of the LDPE/AC composite was measured and reacted with aqueous solutions of the metal ions. Prior to agitation, the pH of the aqueous solutions was meticulously adjusted from 2 to 12 using 0.1 M HCl or NaOH, while all other parameters were held constant. The mixtures were then agitated on a mechanical shaker at a constant speed of 150 rpm and maintained at a controlled temperature of 25 °C. The effect of the initial metal ion concentration was investigated across a range of 0.5 to 10.0 mg/L for both As(III) and As(V). Furthermore, the impact of contact time was examined by measuring the adsorbed metal ions at predefined 5 min intervals ranging from 5 to 50 min. Following agitation, the mixtures were filtered, and the metal ion concentrations in the supernatants were quantified using ICP-OES to ensure the reliability and accuracy of the results. The quantities of adsorbed metal species at equilibrium *Q_e_* and their removal efficiencies %R were calculated using Equations (1) and (2):

(1) $$Q_{e}=\frac{C_{o}-C_{e}}{m}\cdot V$$

(2) $$\%R=\frac{C_{o}-C_{e}}{C_{o}}\cdot 100$$

Here, *Q_e_* is the equilibrium adsorption capacity; *C_o_* and C*_e_* denote the initial and equilibrium concentrations, respectively; V is the volume of the solution; and *m* is the mass of the LDPE/AC composite. The adsorption kinetics were assessed using Equation (3), where *Q_t_* is the adsorption capacity at time *t*, and *C_t_* is the concentration at time *t*. Removal efficiency (*R_t_*) at any time (*t*) was calculated using Equation (4).

(3) $$Q_{t}=\frac{C_{o}-C_{t}}{m}\cdot V$$

(4) $$R_{t}=\frac{C_{o}-C_{t}}{C_{o}}\cdot 100$$

This approach aligns with the growing interest in developing efficient and cost-effective methods for removing heavy metals from water and wastewater [36]. After each experiment, the solid and liquid phases were separated by filtration through 0.45 μm cellulose acetate syringe filters. The residual metal concentrations (*C_e_* or *C_t_*) in the filtrates were determined using ICP-OES. All investigations were performed in triplicate, and mean values were used for subsequent evaluations.

Experimental Parameters:

- Solution pH: 2 to 12
- Initial metal concentration: 0.5–10.0 mg/L
- Contact time: 5–50 min

#### Characterization Method

The surface morphology and chemical functional groups of the LDPE/AC microplastic samples were characterized using Fourier transform infrared (FTIR) spectroscopy on a Spectrum 3 Tri-Range FT-IR Spectrometer (PerkinElmer, Shelton, CT, USA). The analysis employed scanning wavenumbers ranging from 500 to 4000 cm^−1^. X-ray diffraction (XRD) was used to analyze microplastics and qualitatively assess their crystallinity. The Brunauer–Emmett–Teller (BET) surface area and pore-size distribution of the LDPE/AC samples were determined using a Microtrac MRB Belsorp mini surface (Microtrac MRB, Haan, Germany) and pore-size analyzer (University of South Africa, Florida campus, Johannesburg, South Africa). Prior to analysis, approximately 0.5 g of the sample was degassed at 105 °C for 24 h. Subsequently, the pore-size distribution and BET surface area were measured using a nitrogen adsorption–desorption isotherm. Thermal properties of the LDPE/AC were assessed via thermogravimetric analysis (TGA) using EXSAR6000 SII TG/DTA6200 equipment (Seiko Instruments, Chiba, Japan), where approximately 10 mg of the sample was heated in air at a rate of 10 °C·min^−1^ from ambient temperature to 900 °C. Furthermore, the surface morphology and dispersion were examined via scanning electron microscopy (SEM) using a JSM-IT300 InTouch Scope SEM and a Sigma 360 FE-SEM (ZEISS, Oberkochen, Germany) at an accelerating voltage of 15 kV, following gold sputter-coating. Additionally, the concentration of heavy metal ions in the aqueous media was quantified using inductively coupled plasma optical emission spectroscopy (ICP-OES) on an Agilent 720 series spectrometer.

## 3. Results

### 3.1. Fourier Transform Infra-Red (FTIR) and XRD Analysis

**Figure 2:**
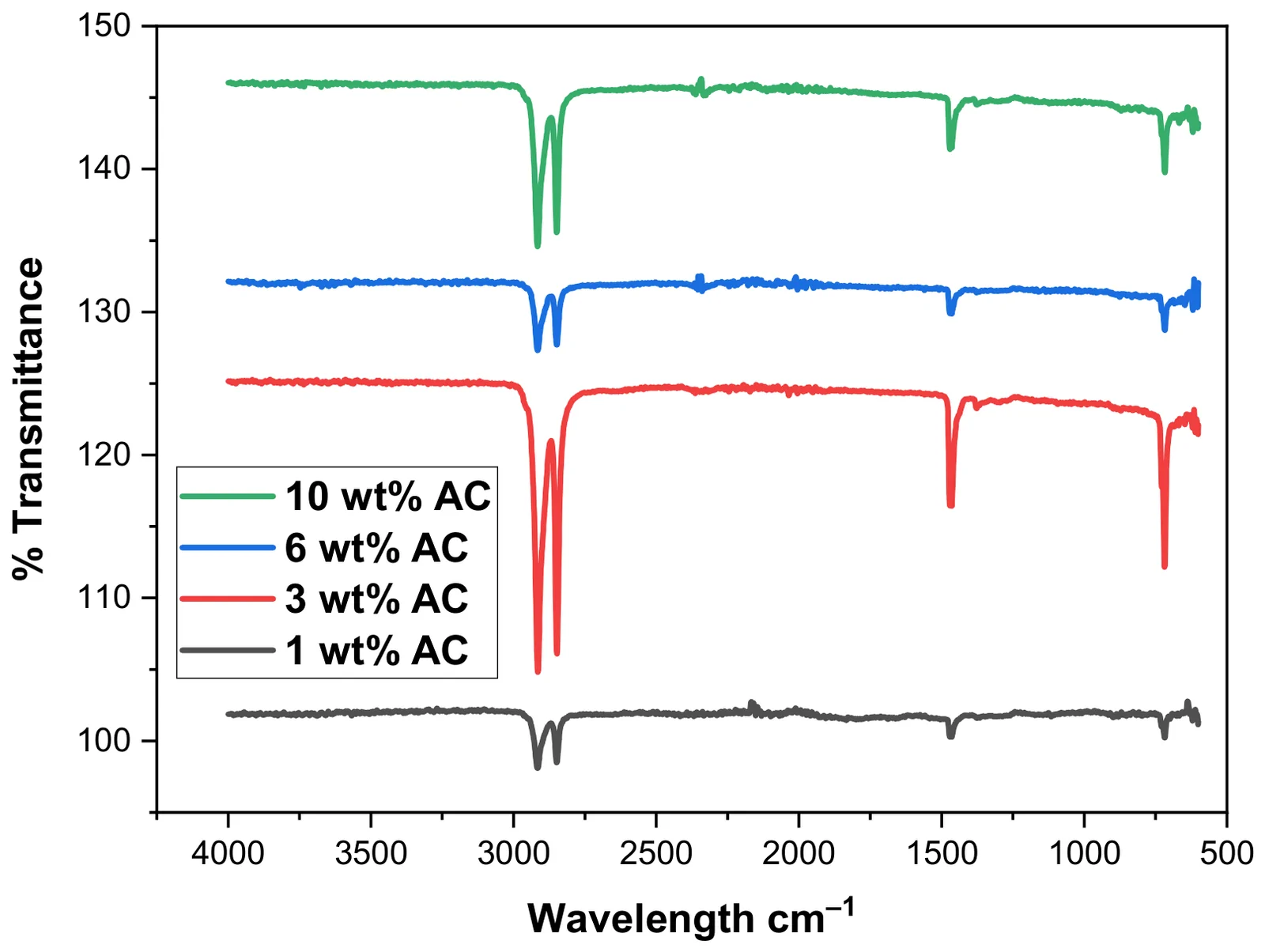
FTIR spectra of LDPE/AC samples.

FTIR spectroscopy was used to identify functional groups and analyze the crystalline structure of the samples based on absorption peak characteristics. Spectra were recorded on a Spectrum 3 Tri-Range FT-IR Spectrometer across the wavenumber range of 500 to 4000^−1^. As shown in Figure 2 and Table 2, characteristic methylene (CH_2_) stretching vibrations appear at 2915 cm^−1^ (asymmetric) and 2849 cm^−1^ (symmetric). Amorphous phases typically exhibit a single CH_2_ rocking peak at ~719 cm^−1^. In contrast, crystalline domains exhibit peak splitting due to interchain interactions—a phenomenon known as crystalline splitting. This splitting is observed in the CH_2_ scissoring region at 1472 and 1464 cm^−1^ and the CH_2_ rocking region at 730 and 720 cm^−1^ [42]. Additionally, a weak band at 1377 cm^−1^ corresponds to the CH_3_ umbrella mode originating from chain end-groups or side branches.

**Figure 3:**
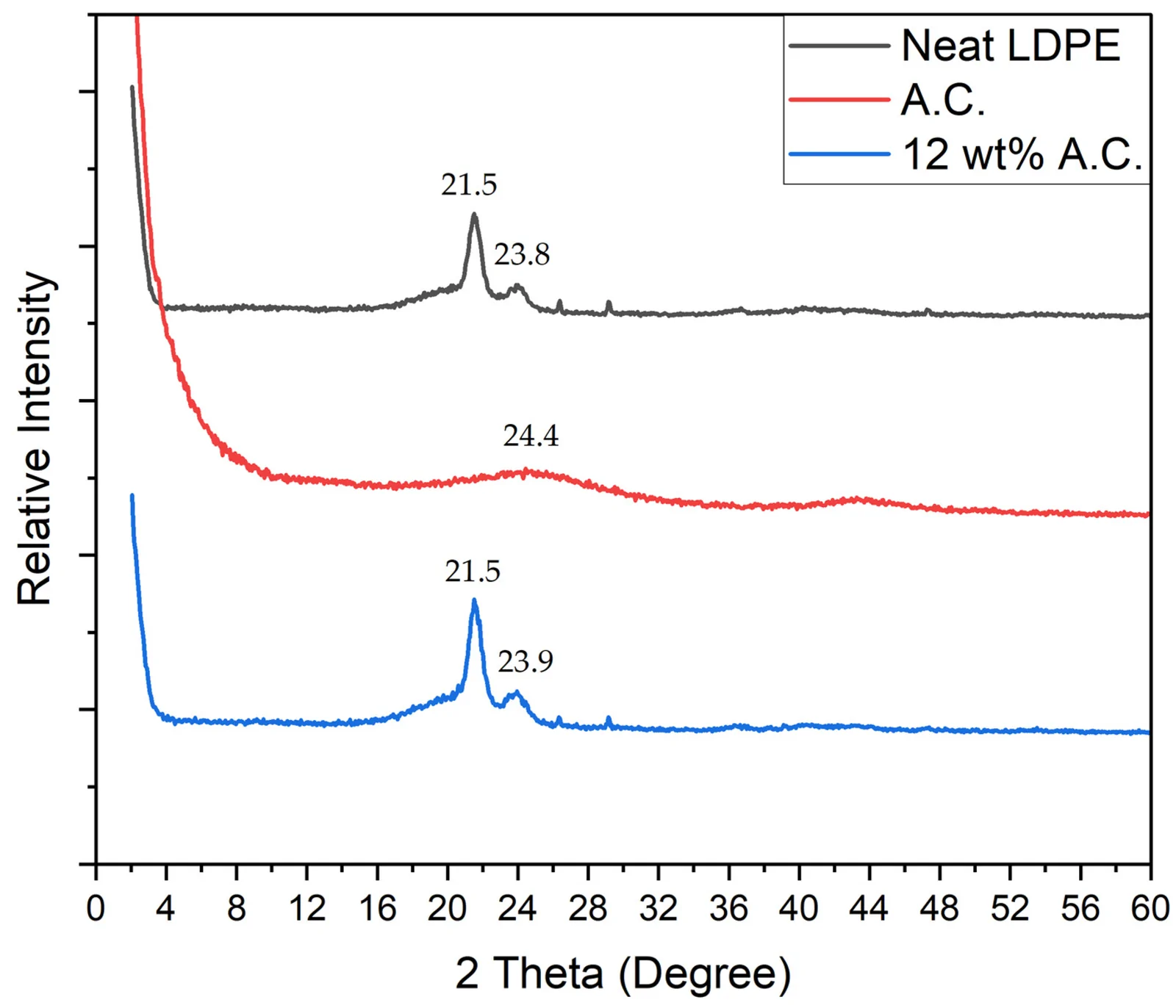
XRD pattern for AC, LDPE, and LDPE/AC 10 wt% composite.

X-ray diffraction (XRD) patterns of the activated carbon (AC), neat LDPE, and the LDPE/AC 10 wt% composite are presented in Figure 3. The diffractogram of neat LDPE displays two distinct crystalline reflections at 2θ 21.5 and 23.8, corresponding to the (110) and (200) crystallographic planes of the orthorhombic polyethylene phase, respectively. LDPE typically exhibits a semi-crystalline structure with 30–50% crystallinity [43] due to short-chain branching that restricts tight chain packing. In contrast, the AC filler exhibits a broad, low-intensity reflection centered at 2θ~24.4, corresponding to the (002) plane of disordered graphitic carbon, confirming its predominantly amorphous nature. The XRD pattern of the LDPE/AC 10 wt% composite retains the main (110) and (200) crystalline reflections of the LDPE matrix, with the broad (002) AC feature overlapping the high-angle shoulder of the (200) peak. The persistence of these sharp reflections indicates that the semi-crystalline matrix structure is preserved upon filler incorporation without significant disruption of the primary polymer crystalline lattice.

### 3.2. Differential Scanning Calorimetry (DSC) Analysis

**Figure 4:**
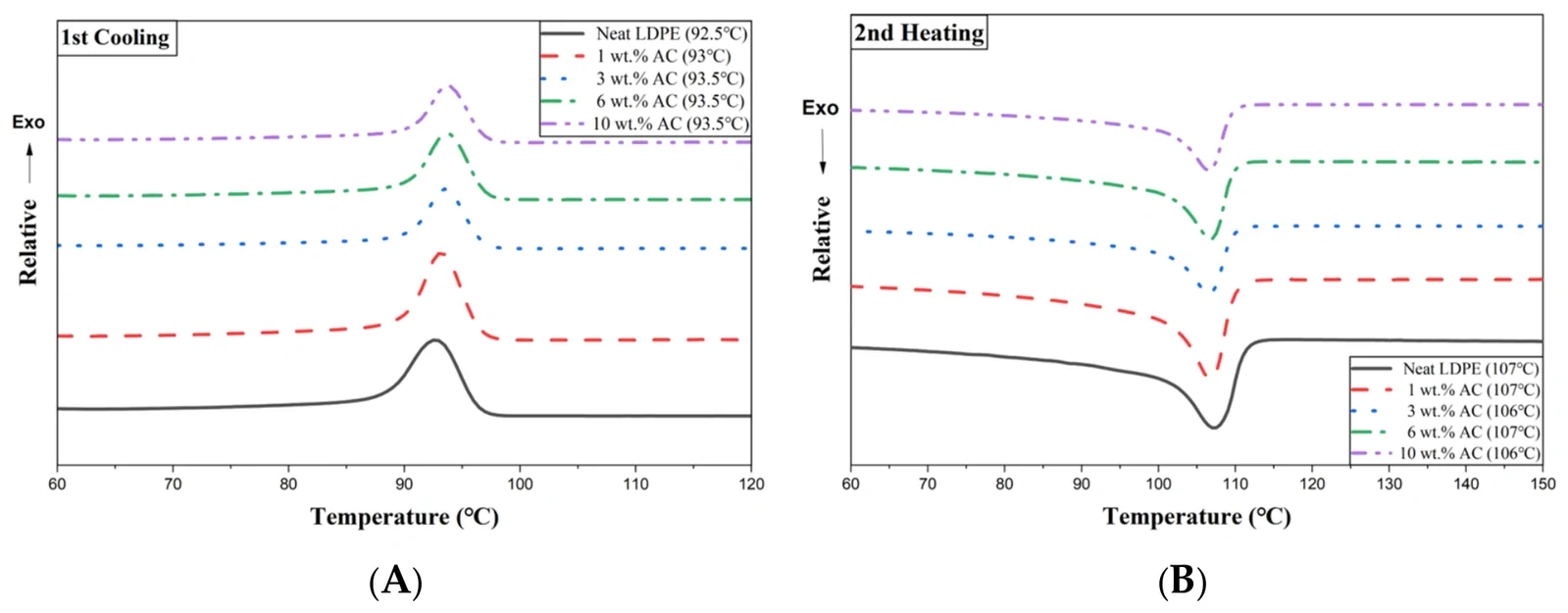
DSC thermograms for (**A**) first cooling and (**B**) second heating scans.

Differential scanning calorimetry (DSC) was used to investigate the thermal transitions and crystallization behavior of neat low-density polyethylene (LDPE) and LDPE/activated carbon (AC) composites containing 1, 3, 6, and 10 wt% AC. The first-cooling and second-heating thermograms are presented in Figure 4. During the second heating scan, neat LDPE exhibited a melting peak at approximately 107 °C. The 1 wt% AC composite showed a similar peak at approximately 107 °C, whereas the 3, 6, and 10 wt% AC composites exhibited melting peaks at approximately 106 °C. This limited variation indicates that the principal melting transition of the LDPE matrix is maintained after AC incorporation. However, the melting temperature alone is insufficient to establish whether the overall degree of crystallinity changes with filler loading. The first-cooling thermograms provide complementary information about crystallization behavior during cooling. The observed crystallization peaks occur at approximately 93 °C based on the plotted traces. Differences in peak position and shape reflect changes in crystallization kinetics associated with AC incorporation. These DSC observations complement the XRD results, which retain the characteristic LDPE reflections at 2 theta values of approximately 21.5° and 23.8°. The combined results indicate that the primary semi-crystalline structure of the LDPE matrix persists after AC incorporation.

### 3.3. Morphological Characterization (SEM and BET Surface Area)

**Figure 5:**
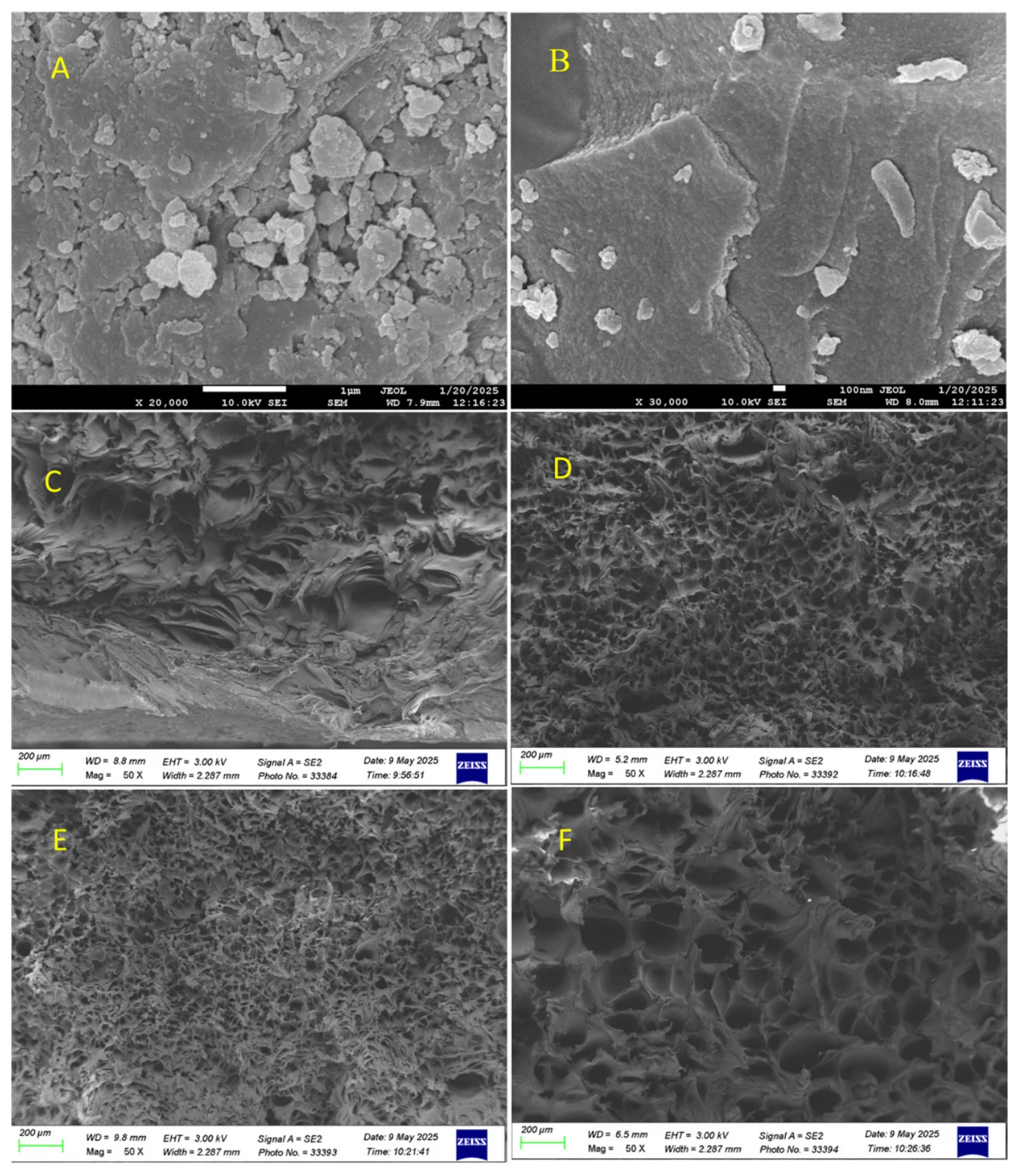
SEM images of Activated carbon (**A**) ×1 µm, (**B**) ×100 nm, and foamed LDPE/AC composites (**C**) 1 wt%, (**D**) 3 wt%, (**E**) 6 wt%, (**F**) 10 wt%.

Figure 5 presents the SEM micrographs of the prepared activated carbon (AC) and the LDPE/AC composites. Images A and B illustrate the surface morphology of the neat AC at high magnification 20,000× and 30,000×, respectively, revealing irregularly shaped, flake-like carbon particles with primary sizes ranging from 1 to 2 μm alongside smaller sub-micron aggregates. Images C through F capture the morphological features of the composite surfaces at lower magnifications: 50× for C–D and 100× for E–F. The composite fracture surfaces display a highly roughened, three-dimensional porous network characterized by ductile tearing and cellular cavities ranging from 50 to 100 μm in width. This structural morphology indicates uniform dispersion of the AC filler throughout the LDPE matrix, with the rough, interconnected feature network contributing to modified mechanical and barrier characteristics.

The Brunauer–Emmett–Teller (BET) specific surface area, pore volume, and average pore radius directly influence physical and chemical properties such as adsorption, catalytic activity, and energy storage capacity. As shown in Table 3, pure activated carbon (AC) exhibits the highest surface area at 1045 m^2^/g [44]. For the LDPE/AC composites, increasing the AC loading from 1 to 10 wt% significantly enhanced the surface area from 134 to 374 m^2^/g (yielding 134, 273, 297, and 374 m^2^/g for 1, 3, 6, and 10 wt%, respectively). Concurrently, both the total pore volume (increasing from 0.397 to 0.455 cm^3^/g) and the average pore radius showed an upward trend with higher AC loading. These findings underscore why optimizing AC content is critical for applications such as toxic heavy metal adsorption, with the 10 wt% composite achieving performance closest to pure AC.

### 3.4. Rheological (Capillary Viscosity) and Thermal (TGA-DTG) Behavior

**Figure 6:**
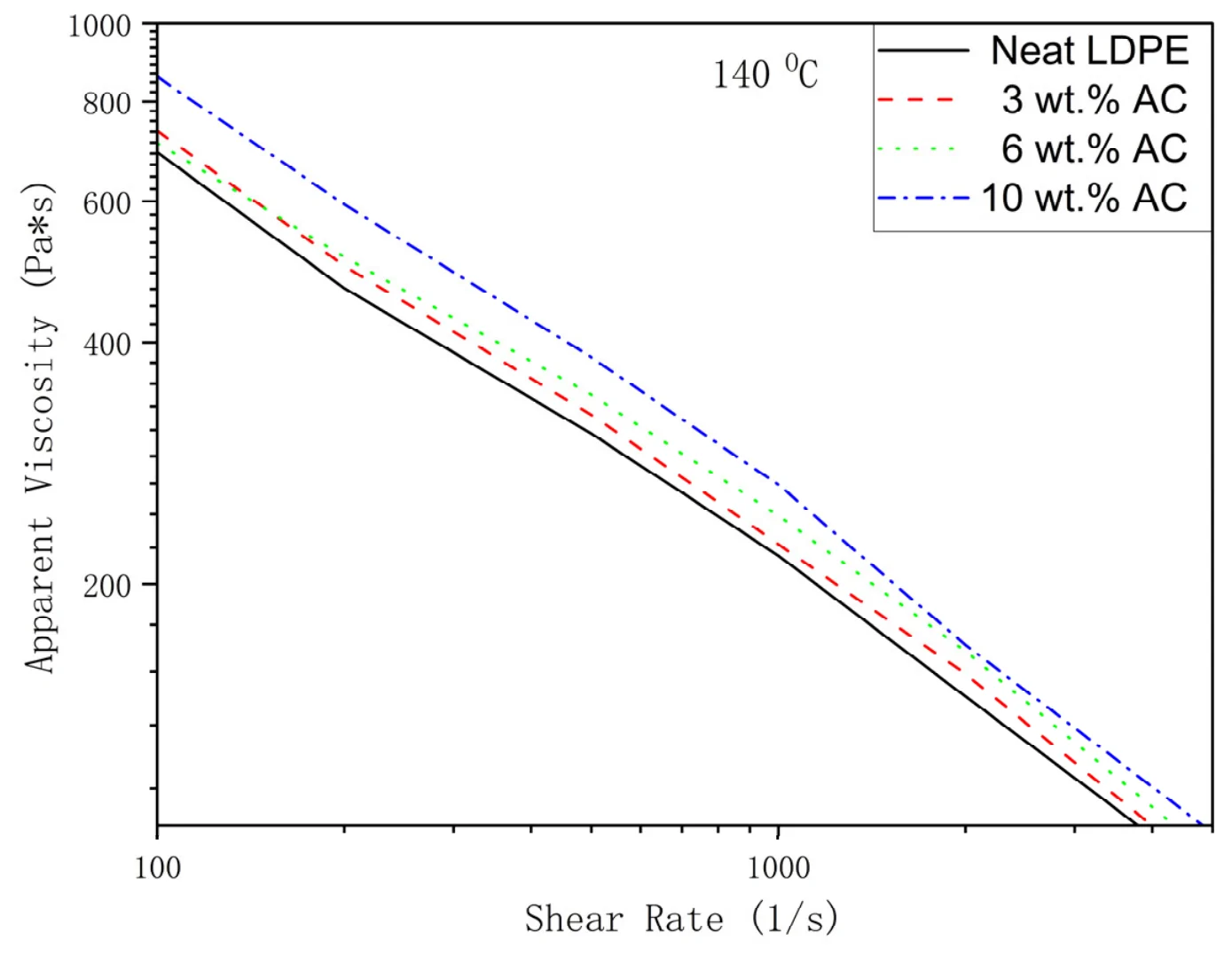
Apparent viscosity of LDPE/AC composites.

The steady-shear viscosity curves for neat LDPE and LDPE/AC composites (3, 6, and 10 wt.%) measured at 140 °C over a shear rate range of 100 to 6000 s^−1^ are shown in Figure 6. All samples demonstrate classic pseudoplastic (shear-thinning) behavior [40], characterized by a monotonic decrease in apparent viscosity with increasing shear rate. At a processing temperature of 140 °C (which is below the thermal decomposition threshold of neat AC), the incorporated blowing agent acts primarily as an un-decomposed solid particulate filler. As the AC concentration increases from 0 to 10 wt.%, the apparent viscosity increases across the entire shear rate spectrum. The solid AC particles restrict the segmental mobility of molten LDPE chains and increase internal dissipation of mechanical energy during shear flow [45].

### 3.5. Thermographic Analysis TGA-DTG

**Figure 7:**
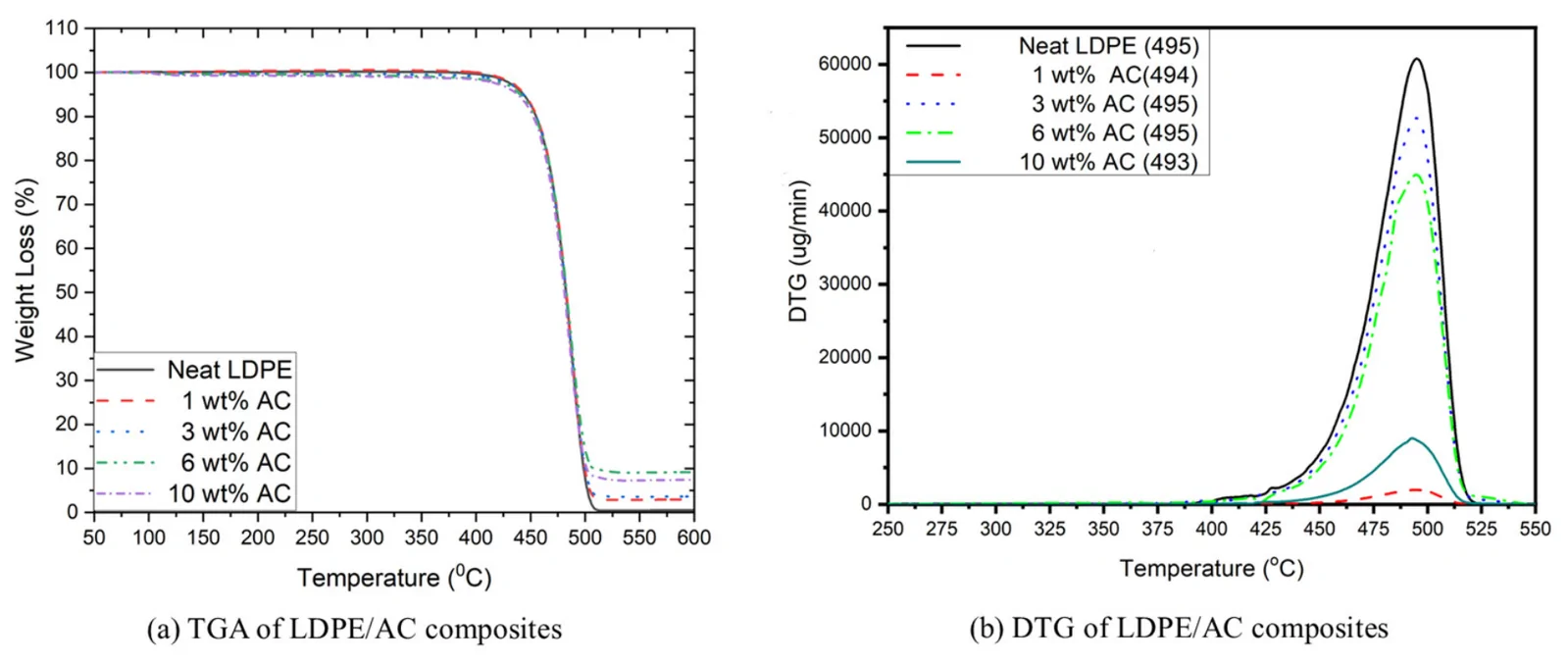
Illustration of (**a**) TGA and (**b**) DTG spectra on the degradation of LDPE/AC composites.

To evaluate the thermal degradation behavior of neat LDPE and the LDPE/AC composites, thermogravimetric analysis (TGA) and derivative thermogravimetric (DTG) curves are presented in Figure 7a and Figure 7b, respectively. Neat LDPE exhibits a literature melting temperature Tm of approximately 108 °C [46], well below its thermal decomposition range. All samples display a single-step degradation profile corresponding to the main-chain scission of the polymer backbone, with complete matrix volatilization occurring below 550 °C. The maximum decomposition temperatures T_max_ derived from the DTG peaks for Neat LDPE and composites containing 1, 3, 6, and 10 wt% AC are 495, 494, 495, 495, and 493 °C, respectively, indicating that the incorporation of AC maintains the intrinsic thermal stability of the polymer matrix. As shown in Figure 7a, the residual mass above 550 °C increases proportionally with filler loading (yielding approximately 1, 3, 6, and 10 wt% char), corresponding to the high thermal stability of the graphitic carbon framework under the tested temperature range.

### 3.6. Mechanical Performance (Tensile Testing)

**Figure 8:**
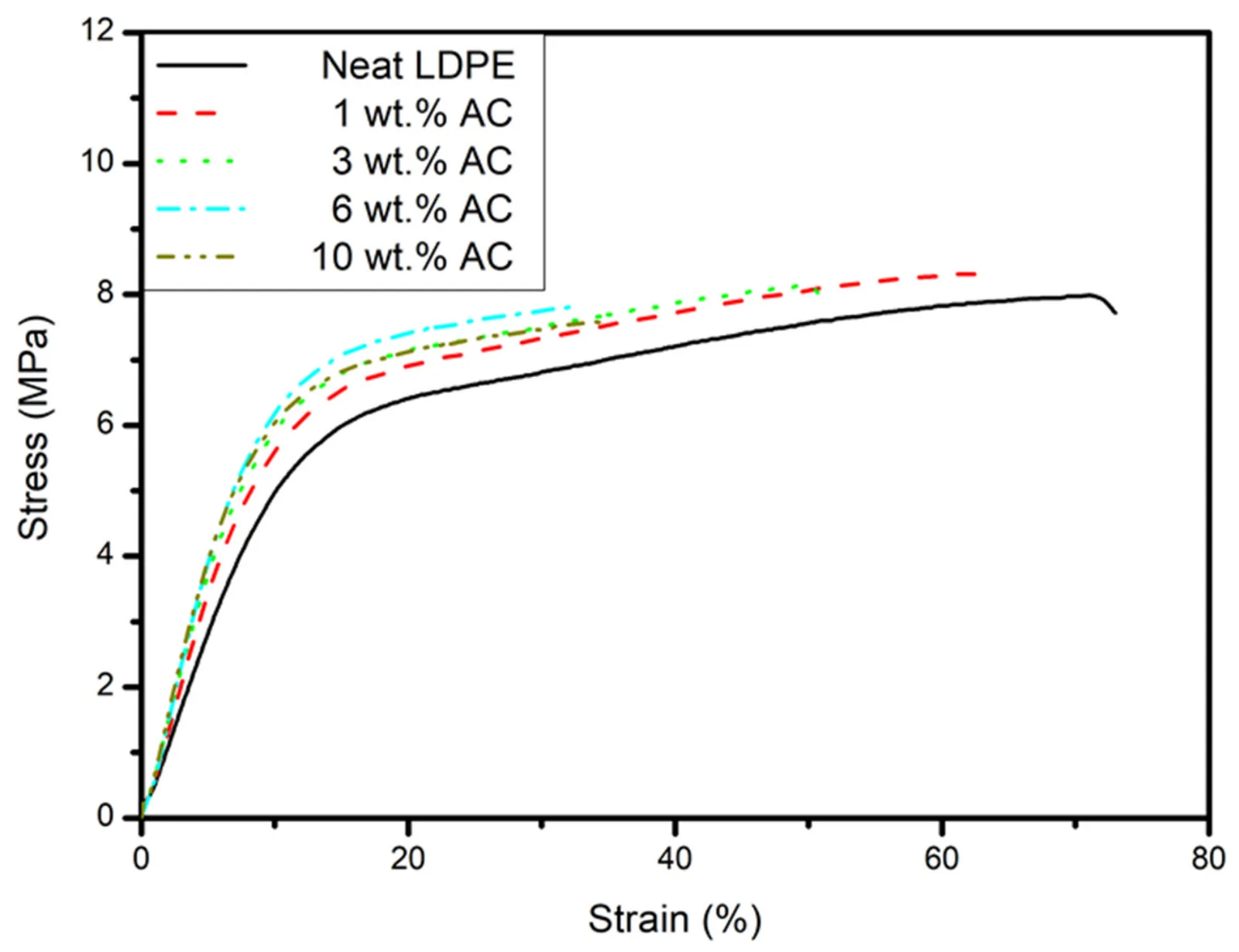
Foamed tensile strength of LDPE/AC composites.

The uniaxial tensile stress–strain curves of neat low-density polyethylene (LDPE) and LDPE/AC composites with varying AC contents (1, 3, 6, and 10 wt.%) are illustrated in Figure 8. The mechanical parameters, including initial elastic modulus, peak stress, and elongation at break, demonstrate a strong dependence on the blowing agent concentration and the resulting cellular morphology (Table 4).

As depicted in Figure 8, increasing the AC content leads to a progressive increase in the initial linear slope of the stress–strain curves, indicating an enhancement in stiffness (tensile modulus) relative to neat LDPE. Neat LDPE exhibits the lowest initial slope. Upon the addition of 1 wt.% to 10 wt.% AC, the apparent elastic modulus increases systematically, reaching its maximum at 6–10 wt.% AC. This enhancement in initial stiffness can be attributed to the introduction of a fine cellular micro-structure and undecomposed blowing agent particles acting as nucleating centers. Under low-strain deformation, the cell walls restrict macro-molecular chain relaxation, thereby increasing the initial resistance to elastic deformation. At low AC loading (1–3 wt.%), the peak tensile stress slightly increases from ~8.0 MPa (neat LDPE) to ~8.3 MPa (1 wt.% AC) and ~8.1 MPa (3 wt.% AC). At these lower concentrations, well-dispersed micro-voids and uniform cell growth maintain effective stress transfer across the polymer matrix without severely compromising structural integrity. While at high AC loading (6–10 wt.%), a modest decline in peak stress is observed, dropping to ~7.8 MPa for 6 wt.% AC and ~7.4 MPa for 10 wt.% AC. This strength reduction stems from an increased void volume fraction (higher overall porosity) and cell wall thinning, which reduces the effective load-bearing cross-sectional area of the polymer matrix. Aussawasathiena et al. [32] reported that neat LDPE has the largest tensile strength of foamed LDPE/AC composites with the chemical blowing agent azodicarbonamide. Unlike stiffness, the elongation at break decreases monotonically with increasing AC concentration. Neat LDPE demonstrates characteristic ductile behavior, achieving a maximum strain at break of ~73%. With progressive AC addition, the failure strain drops significantly to ~63%, ~52%, ~33%, and ~31% for 1, 3, 6, and 10 wt.% AC, respectively.

### 3.7. Zeta Potential for Ac and LDPE/AC Composite

**Figure 9:**
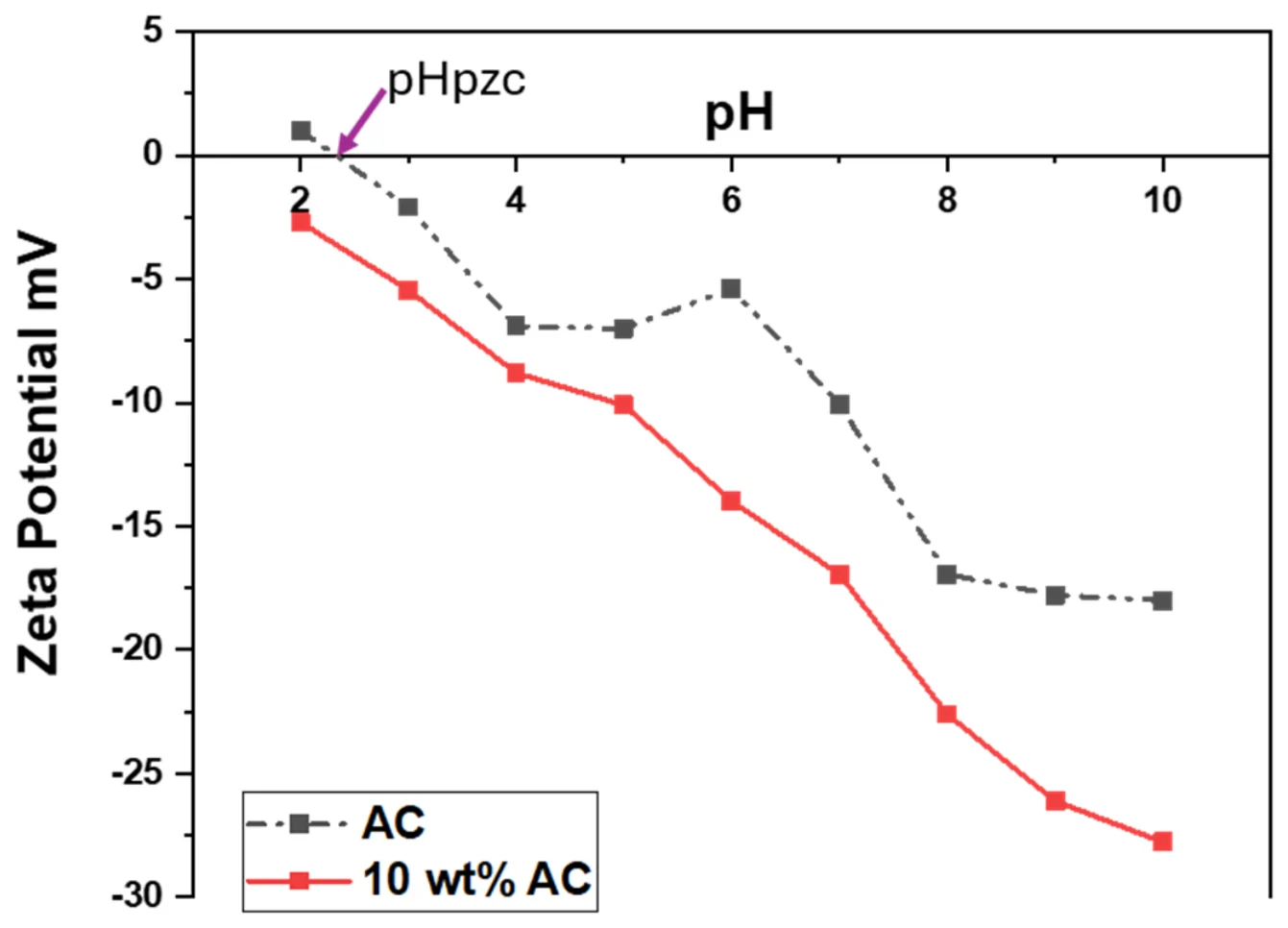
Zeta potential (mV) curve of AC and 10 wt. %AC/LDPE at various pH values.

pH plays an important role in adsorption because it affects the surface charges of both the adsorbent and the contaminant. The effect of pH on As(III) and As(V) remediation. As(III) remediation increased as the pH rose from 2.0 to 9.0, then stabilized between 9.0 and 14.0. In contrast, As(V) remediation showed an inverse relationship with pH: removal was highest initially and declined as pH increased. Figure 9 compares the zeta potentials (zeta) of AC and LDPE/AC across different pH values, showing how surface-charge interactions influence adsorption between pH 2 and 10. The point of zero charge (pHpzc) is at pH 2.3, and both AC and LDPE/AC are negatively charged above this value. Therefore, electrostatic interactions alone cannot explain the removal of As(III) and As(V) by the LDPE/AC composite. Instead, these results suggest that hydrogen bonding may contribute to adsorption, given the presence of polar groups such as –COOH and –OH. As(III) and As(V) can form hydrogen bonds with these functional groups, enhancing their adsorption and transport in the media.

### 3.8. Arsenic [As(III) & As(V)] Adsorption Studies and Mechanisms

#### 3.8.1. Effect of Initial Concentration

**Figure 10:**
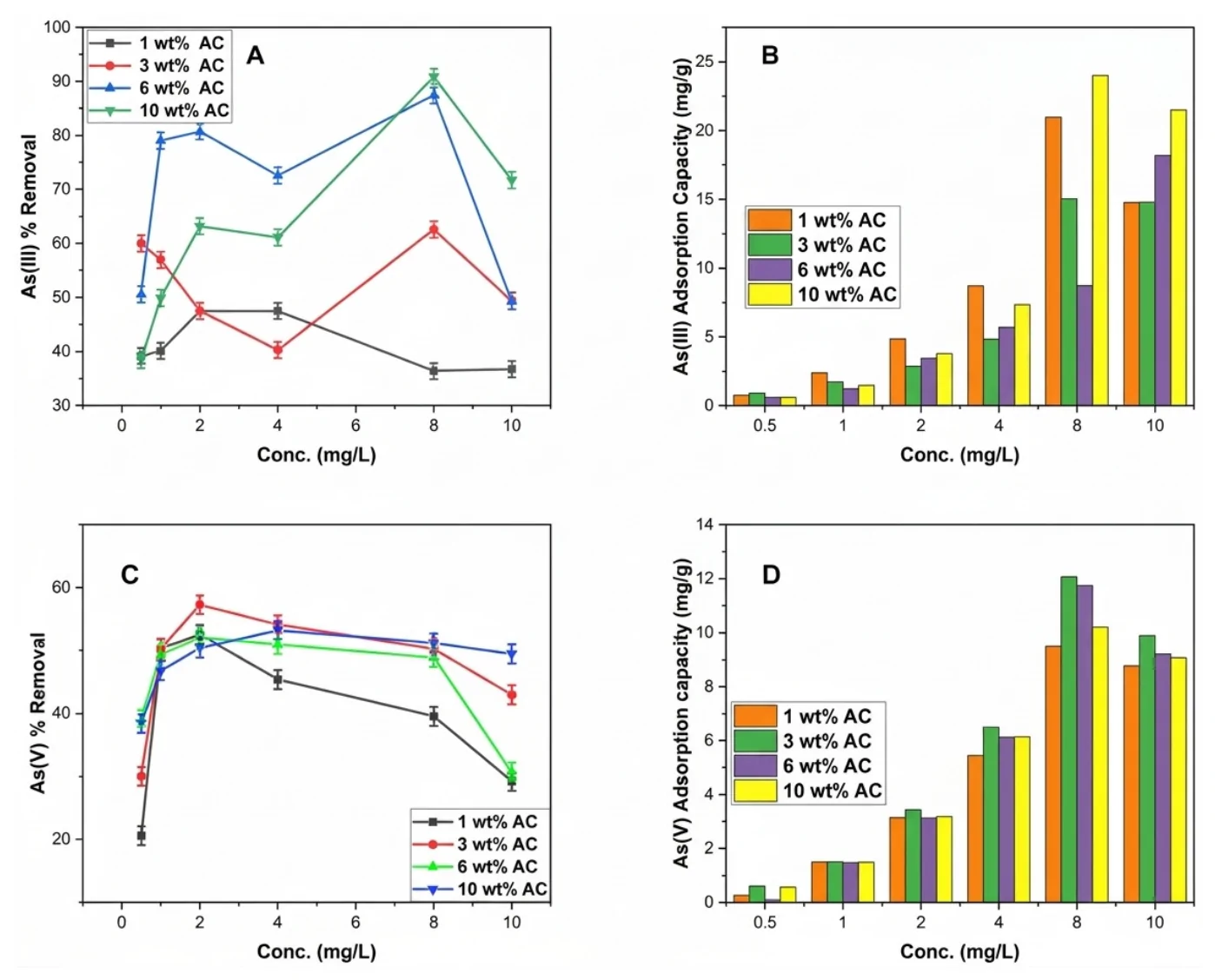
Illustration of initial concentration percentage and capacity removal of As(III) and (V) using AC/LDPE, where (**A**) removal % of As(III), (**B**) adsorption capacity of As(III), (**C**) removal % of As(V) and (**D**) adsorption capacity of As(V).

The effect of initial metal ion concentration (0.5 to 10 mg/L) on the adsorption performance of As(III) and As(V) using LDPE/AC composites (1, 3, 6, and 10 wt% AC) is illustrated in Figure 10. Plots A and C present the percentage removal for As(III) and As(V), respectively, while Plots B and D show the corresponding adsorption capacities (mg/g).

For As(III), performance improved significantly with higher AC loading, with the 10 wt% AC composite exhibiting the highest removal efficiency (91%) and maximum adsorption capacity (24 mg/g) at an initial concentration of 8 mg/L (Figure 10A,B). For As(V), the maximum removal efficiency reached approximately 57% using the 3 wt% AC composite at 2 mg/L (Figure 10C), while peak adsorption capacities of 10.2 mg/g for 10 wt% AC and ~12 mg/g for 3 wt% and 6 wt% AC were attained at 8 mg/L (Figure 10D). Overall, higher AC loading markedly enhances As(III) uptake, whereas As(V) adsorption demonstrates optimal performance at lower to moderate AC ratios [47].

#### 3.8.2. Effect of pH and Contact Time

**Figure 11:**
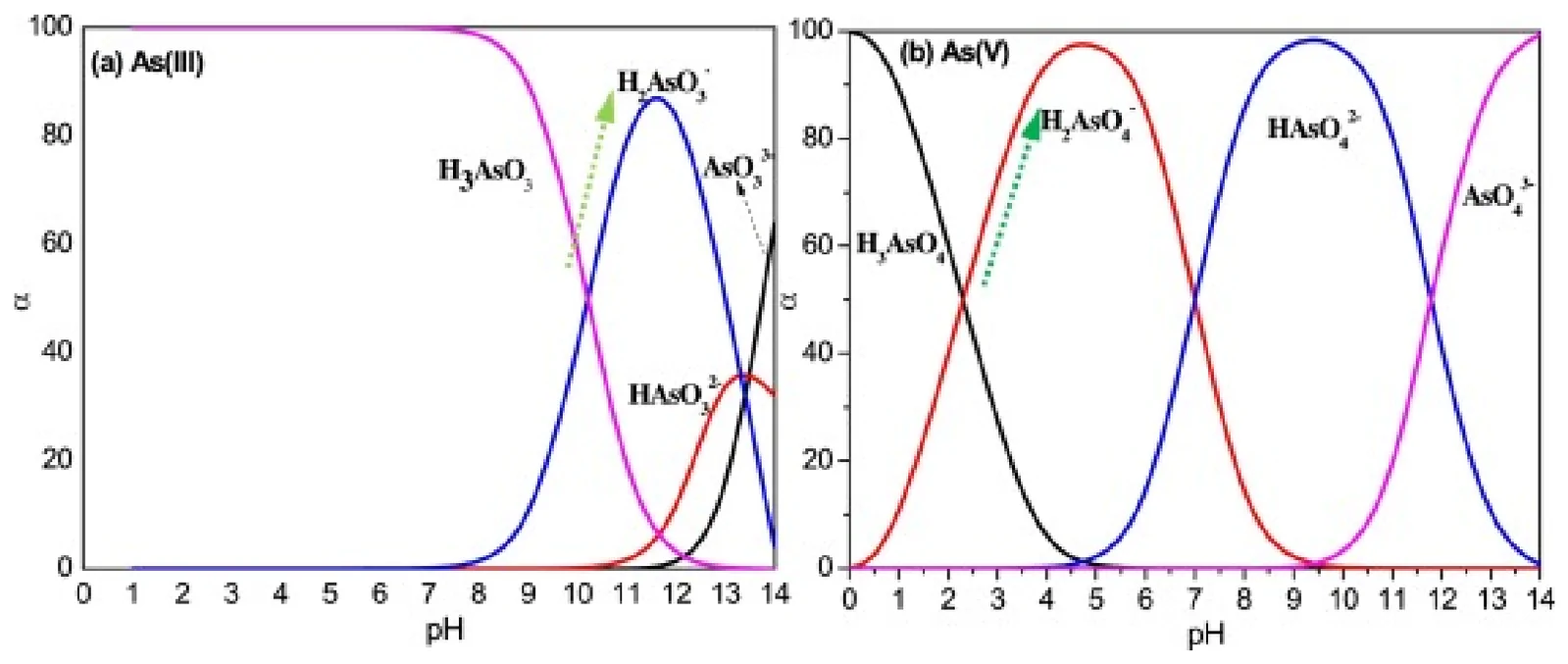
Distribution of (**a**) As(III) and (**b**) As(V) in solution [40].

**Figure 12:**
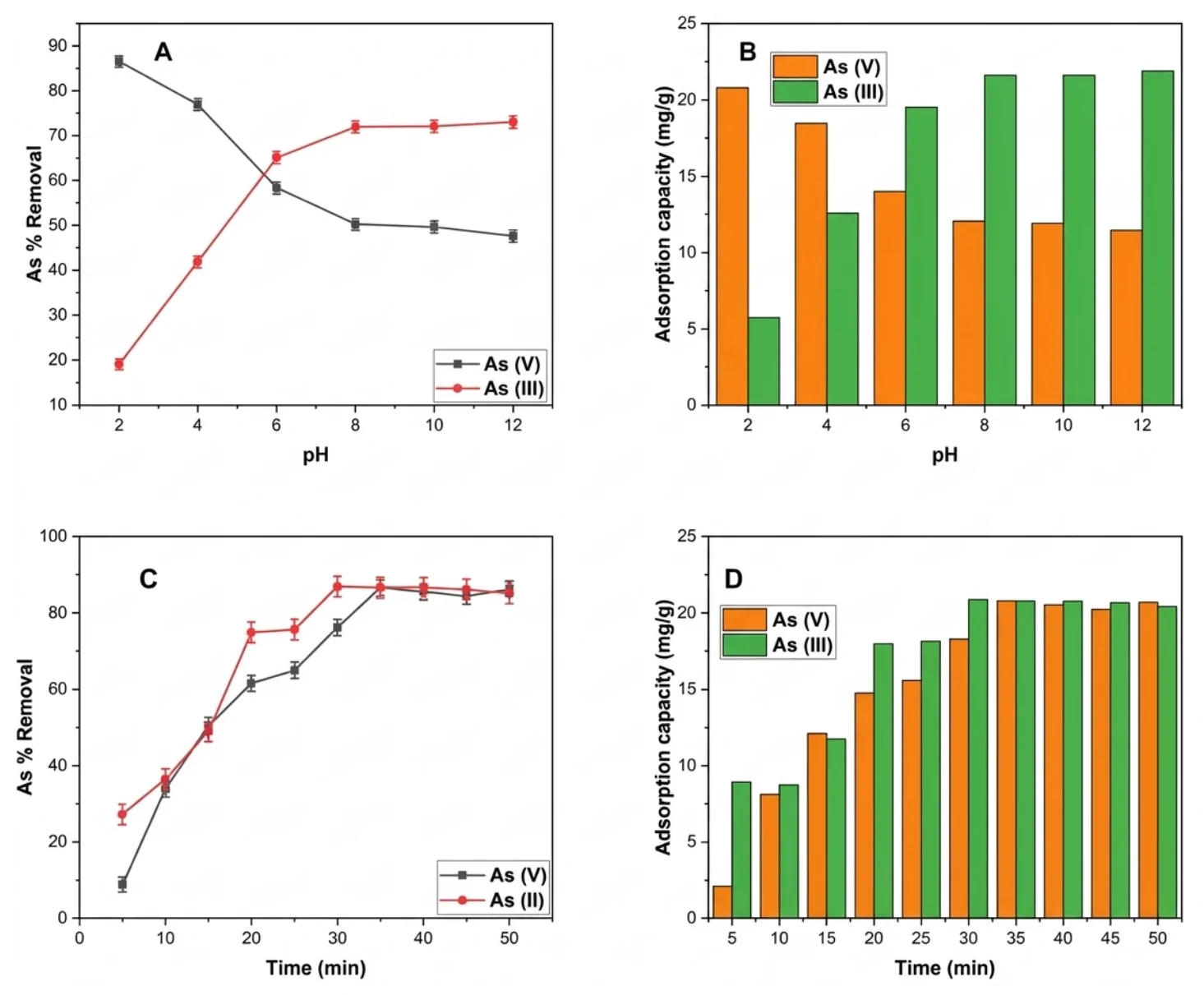
Illustration of pH and time dependence percentage and capacity removal of As(III) and (V) using LDPE/AC, where (**A**) removal % of As(III), (**B**) adsorption capacity of As(III), (**C**) removal % of As(V), (**D**) adsorption capacity of As(V).

The pH of the solution is a critical parameter in adsorption processes, as it governs the surface charge of the adsorbent, the protonation level of functional groups, and the chemical speciation of arsenic species in aqueous solution. As shown in Figure 11, As(III) exists predominantly as the neutral species H_3_AsO_3_ below pH 9, which dissociates into anionic species such as H_2_AsO_3_ and HAsO_3_^2−^ (Figure 11a). In contrast, As(V) exists mainly as monovalent H_2_AsO_4_^−^ between pH 3 and 6, and divalent HAsO_4_^2−^ between pH 7 and 11 (Figure 11b). This chemical speciation directly dictates adsorption behavior across different pH regimes (Figure 11). Below pH 3, competitive adsorption with H_3_O^+^ ions suppresses arsenic uptake, whereas H_3_O^+^ surface interactions and ion exchange become favorable in the intermediate pH range [48]. Maximum removal efficiency for As(III) was achieved near pH 7–8, where competition is minimized, whereas As(V) removal peaked under acidic conditions (pH ~3), where the adsorbent surface is positively protonated, facilitating electrostatic attraction with H_2_AsO_4_^−^. Contact-time experiments with 0.5 g of the 10 wt% AC composite (8 mg/L initial concentration; pH 3 for As(V) and pH 8 for As(III)) showed rapid kinetics (Figure 12C,D). Optimum removal efficiencies reached 88% for As(V) and 86% for As(III), corresponding to an adsorption capacity of approximately 20.71 mg/g for both species [49]. Adsorption equilibrium was achieved within 25 to 30 min, beyond which no significant further uptake occurred.

### 3.9. Adsorption Mechanism

**Scheme 1:**
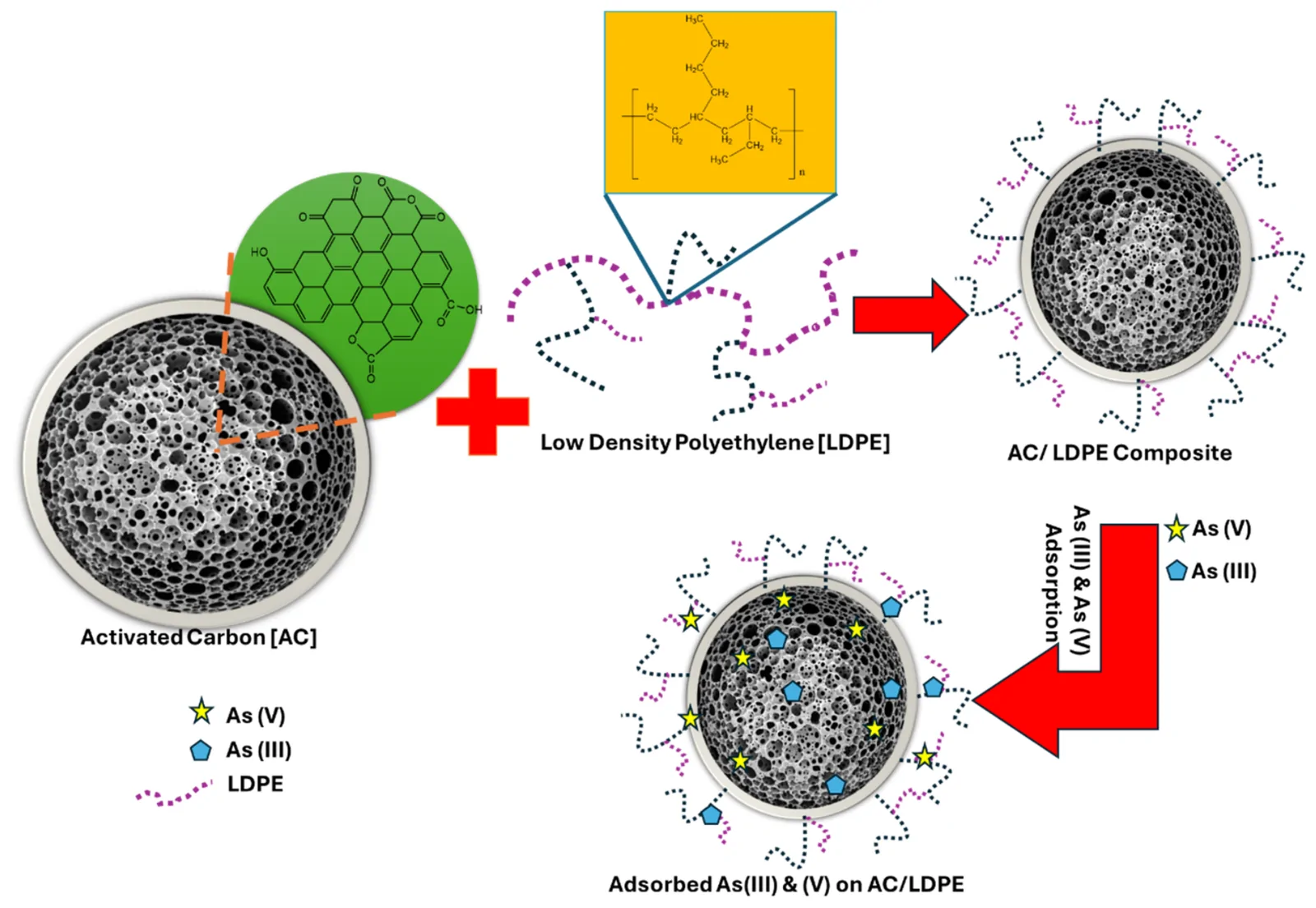
Mechanism illustrating the adsorption of As(III)& (V) using LDPE/AC.

The adsorption of heavy metals onto activated carbon (AC) functionalized within low-density polyethylene (LDPE) matrices is a complex process influenced by aqueous characteristics and physicochemical interactions as shown in Scheme 1. The adsorption of arsenic species by LDPE/AC composites is influenced by surface charge, aqueous speciation, accessible surface functional groups, and pore structure. The zeta potential results indicate that both AC and LDPE/AC surfaces are negatively charged above the reported point of zero charge (pH_p_zc = 2.3; Figure 9). However, the pH-dependent adsorption behavior suggests that electrostatic interactions alone cannot account for arsenic removal. As(III) predominantly exists as neutral H_3_AsO_3_ below approximately pH 9 (Figure 11a), yet its removal increased with increasing pH. This trend suggests that additional factors, potentially including interactions with accessible surface functional groups and changes in adsorption-site availability, contribute to As(III) uptake. In contrast, As(V) removal was highest under acidic conditions and decreased as pH increased, indicating that arsenic speciation and pH-dependent surface interactions influence its adsorption. The higher surface area and porous morphology indicated by BET analysis (Table 3 and Figure 5) are consistent with a potential contribution from pore adsorption.

### 3.10. Kinetics Model

The kinetics of heavy metal adsorption provide insight into the reaction pathways and the mechanism of rate-controlling steps. To evaluate the adsorption dynamics of As(III) and As(V) onto the 10 wt% AC composite, two classic kinetic models were applied.

1. Pseudo-First-Order (PFO) Model

The pseudo-first-order model, also known as the Lagergren model, assumes that the rate of adsorbate uptake over time is directly proportional to the difference between the equilibrium adsorption capacity and the amount adsorbed at any given time. It is widely used to describe the initial stages of adsorption. The linear form of the Lagergren PFO equation [50] is expressed as:

(5) log(qe−qt)=log   qe−K12303 t

where *q_e_* and *q_t_* represent the amounts of As(III) and As(V) adsorbed onto the composite at equilibrium and at any given time t, respectively, and K1 represents the pseudo-first-order rate constant min^−1^.

**Figure 13:**
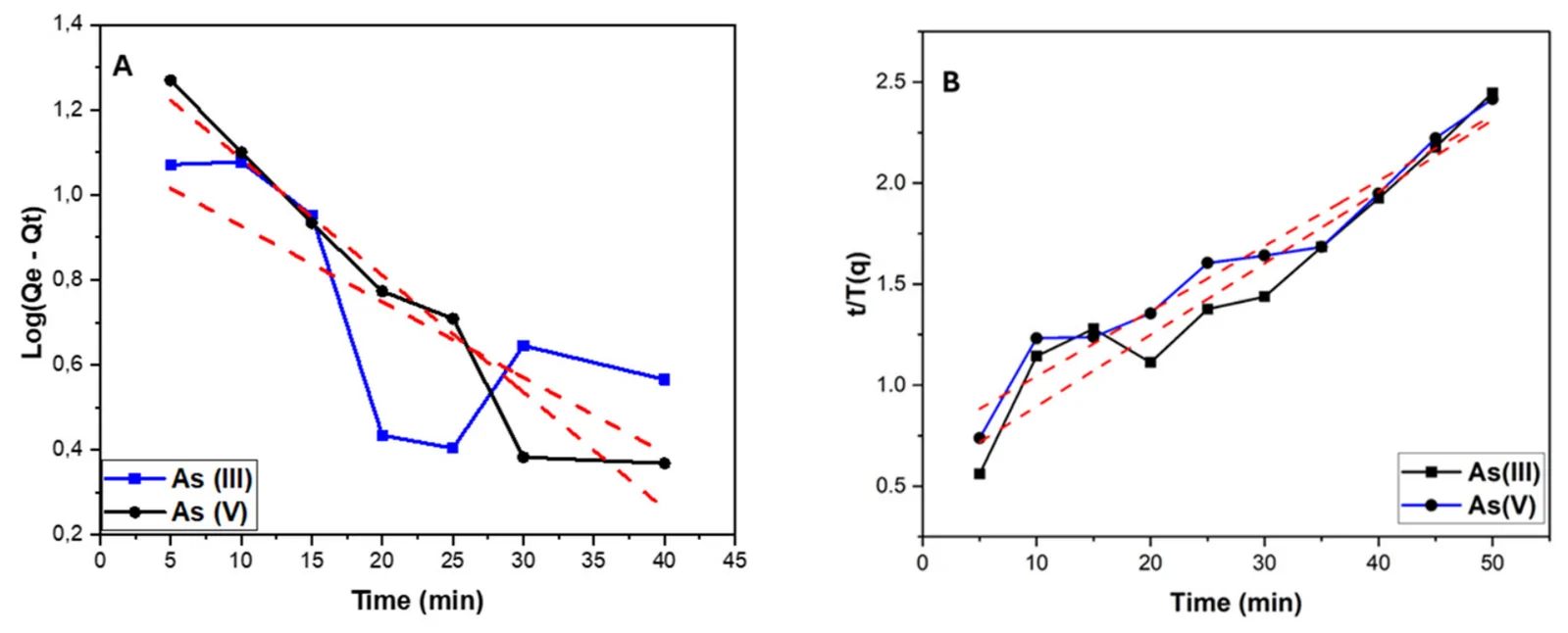
Linearized fits for pseudo-first (**A**) of As(III) and As(V), 0.5g 10 wt% AC polymer composite (**B**). Linearized fits for a second order of As(III) and As(V), 0.5g 10 wt% AC composite, at 25 °C.

The rate constant and correlation coefficients for various initial concentrations were calculated from the linear plots of log *q_e_* − *q_t_* versus t (as illustrated in Figure 13A). The high coefficients of determination R^2^ suggest that the initial stages of As(III) and As(V) uptake on the 10 wt% LDPE/AC composites align closely with physical adsorption characteristics. Figure 13A illustrates this linear plot for the pseudo-first-order model. The linear fits, with coefficients of determination (R^2^) of 0.896 and 0.868, indicate that the PFO model is not favorable.

2. Pseudo-Second-Order (PSO) Model

The pseudo-second-order kinetic model describes the relationship between adsorption capacity and time, reflecting the availability of active surface sites. Unlike the first-order model, it assumes that the rate-limiting step is chemisorption, involving valence forces through the sharing or exchange of electrons between the adsorbent and adsorbate. Typically, the applicability of the PSO model is evaluated using the coefficient of determination, where values close to 1 (R^2^ > 0.9) indicate a strong fit. The linear form of the pseudo-second-order kinetic equation, as described by Tran [51], is expressed as:

(6) $$\frac{t}{q_{t}}=\frac{1}{K_{2}q_{e}^{2}}+\frac{1}{q_{e}}\;t$$

where *q_e_* and *q_t_* (mg/g) represent the adsorption capacities at equilibrium and at time t (min), respectively, and *K*_2_ (g/(mg·min)) is the pseudo-second-order rate constant. It should be noted that the pseudo-second-order kinetic model is applicable only if the plot of *t*/*q*(*t*) versus t exhibits a linear relationship [52].

Figure 13B illustrates this linear plot for the pseudo-second-order model. The linear fits, with coefficients of determination of R^2^ = 0.916 and 0.947 for As(III) and As(V), respectively (as summarized in Table 5), indicate that the PSO model is favorable, reflecting the pseudo-second-order nature of As(III) and As(V) adsorption onto the 10 wt% LDPE/AC composite. Consequently, this kinetic behavior complements the equilibrium data, which demonstrated that the Freundlich isotherms fit the experimental data for As(III) and As(V) removal, as indicated by their respective R^2^ values [53]. The comparison of different parameters from literature on activated carbon/low-density polyethylene for removing As(III) and As(V) from aqueous media is shown in Table 6.

### 3.11. Adsorption Isotherm

#### 3.11.1. Langmuir Isotherm

The Langmuir isotherm model assumes monolayer adsorption onto a surface with a finite number of identical, homogeneous active sites. The non-linear expression is given by:

(7) $$q_{e}=\;\frac{q_{m}\;K_{L}\;C_{e}}{1+{\;K_{L}C}_{e}}$$

The linearized form can be expressed as:

(8) $$\frac{C_{e}}{q_{e}}=\;\frac{1}{q_{m}\;K_{L}}+\frac{C_{e}}{q_{m}}$$

where *q_e_* is the equilibrium adsorption capacity (mg/g), *q_m_* is the maximum monolayer capacity (mg/g), *C_e_* is the equilibrium adsorbate concentration (mg/L), and *K_L_* is the Langmuir constant related to adsorption energy (L/mg).

**Figure 14:**
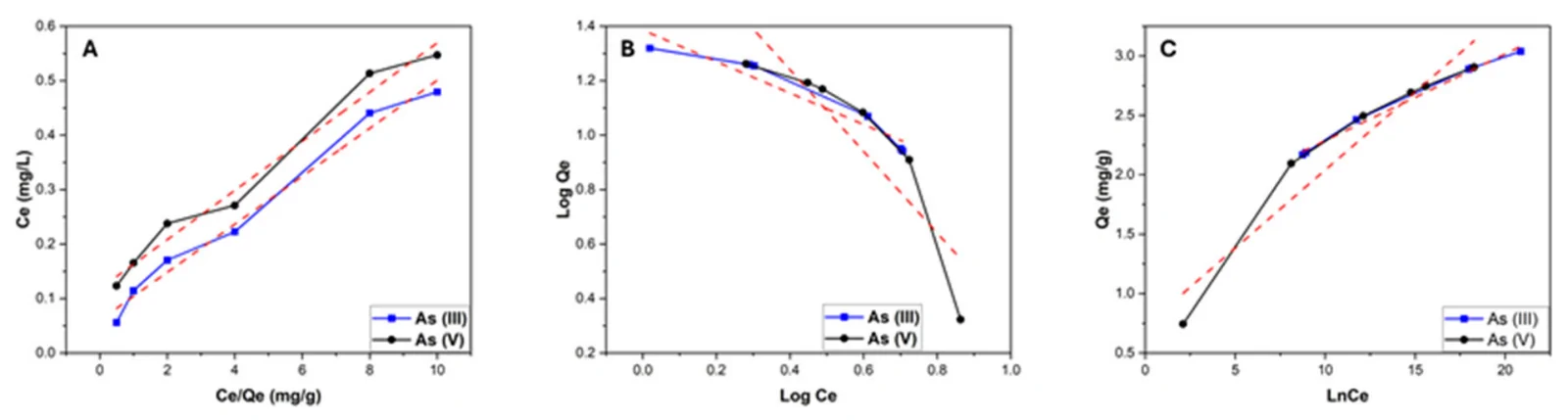
Linearized fits for (**A**) Langmuir, (**B**) Freundlich, and (**C**) Temkin isotherms with As(III) and As(V), 0.5 g, 10 wt% AC composite.

When plotting *C_e_*/*q_e_* against *C_e_* (Figure 14A), the linear correlation coefficient (R^2^ = 0.98) indicates the degree of fit to the Langmuir model for the 10 wt% AC composites.

An essential parameter of the Langmuir model is the dimensionless separation factor, *R_L_*, which indicates whether the process is favorable, as defined by:

(9) $$\mathrm{RL}=\frac{1}{1+K_{L}\;C_{o}}$$

where *C_o_* is the initial adsorbate concentration (mg/L). As summarized in Table 7, an *RL* value between 0 and 1 indicates favorable adsorption. In this study, the calculated R_L_ values ranged from 0.35 to 0.73, confirming that adsorption of As(III) and As(V) onto the composite is favorable.

#### 3.11.2. Freundlich Isotherm

The Freundlich isotherm describes adsorption onto a heterogeneous surface with active sites of varying adsorption energies. The non-linear and linearized forms are expressed as [60]:

(10) $$q_{e}=\;K_{f}C_{e}^{1/n}\to \log q_{e}=\log K_{f}+\frac{1}{n}\log C_{e}$$

where *K_f_* is the Freundlich affinity coefficient [(mg/g)(L/mg)^1/*n*^] and 1/*n* is the heterogeneity factor. The linear plot of log *q_e_* versus log *C_e_* is shown in Figure 14B. The linear plots exhibited high correlation coefficients (R^2^= 0.92) (Table 8), indicating the suitability of the Freundlich isotherm model for describing the adsorption process on the 10 wt% LDPE/AC composites for both As(III) and As(V) uptake.

#### 3.11.3. Temkin Isotherm

The Temkin model assumes that the heat of adsorption decreases linearly with surface coverage due to interactions between the adsorbent and the adsorbate (metal ions/dye molecules). This model is expressed by Equation (11) below:

*q_e_* = B *In K_T_* + B *In C_e_* (11)

where B = RT/b_T_ is a constant related to the heat of adsorption (J/mol), R is the universal gas constant (8.314 J/(mol·K)), T is the absolute temperature (K), *K_T_* is the Temkin isotherm equilibrium binding constant (L/g), q_e_ is the amount of arsenic adsorbed at equilibrium (mg/g), and *C_e_* is the equilibrium arsenic concentration (mg/L) [61]. The constants B and *K_T_* can be determined from the slope and intercept of the linear plot of *q_e_* versus *LnCe*, as illustrated in Figure 14C.

#### 3.11.4. Intraparticle Diffusion Model

**Figure 15:**
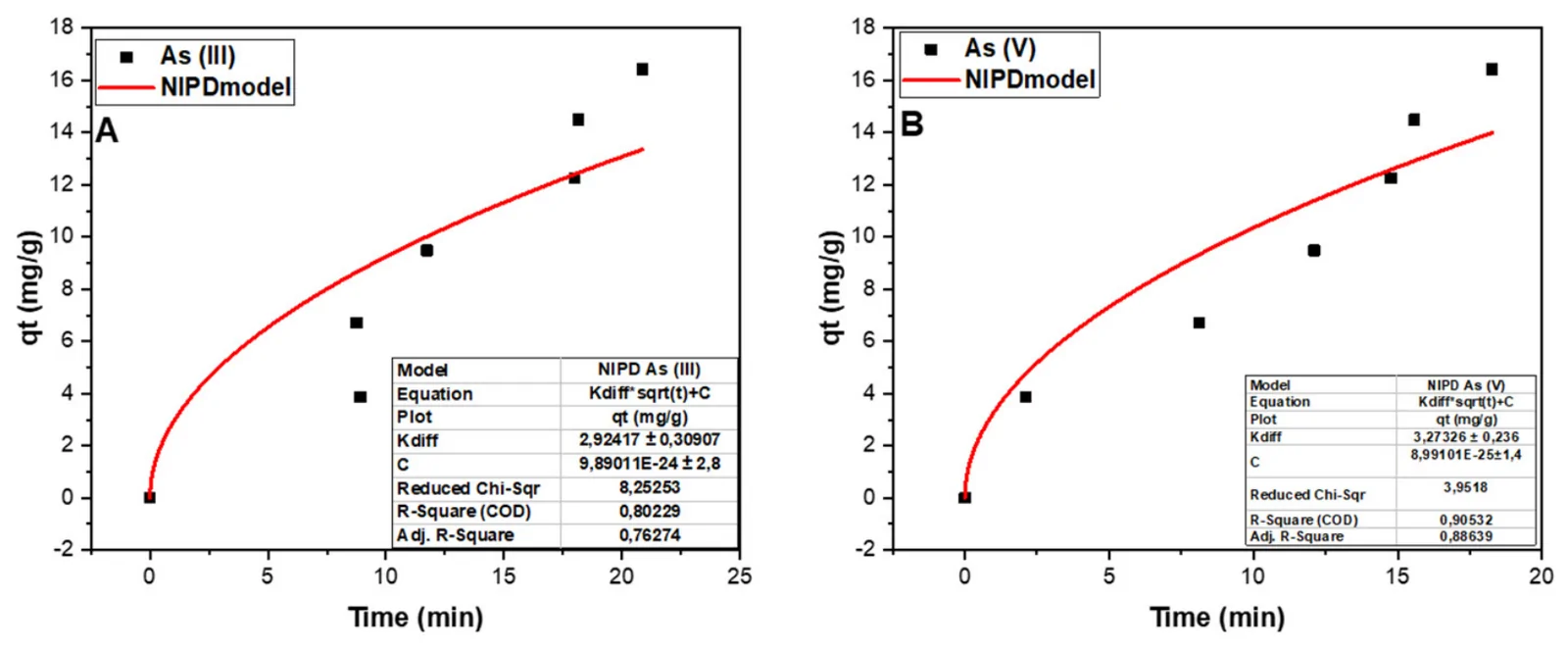
Plots of McKay and Poots intraparticle diffusion model for the adsorption of As(III) and As(VI) onto 10 wt% AC, where (**A**) As(III) NIPD model and (**B**) As(VI) NIPD model onto 10 wt% AC.

The McKay–Poots model for intraparticle diffusion was used to describe the adsorption of As(III) and As(V) onto 10 wt% LDPE/AC. This model describes the solute diffusion within porous adsorbents under the premise that intraparticle diffusion is the rate-limiting step, with its rate being proportional to the concentration gradient within the particle. According to the model, a plot of solute uptake (*q_t_*) versus the square root of time (t^0.5^) yields a straight line when intraparticle diffusion is involved in the sorption process. If the linear regression passes through the origin, intraparticle diffusion is the sole rate-limiting step. However, if the plot exhibits a non-zero intercept, intraparticle diffusion is not the sole rate-controlling mechanism, indicating that surface or boundary-layer diffusion may operate simultaneously.

(12) $$q_{t}=k_{1}t^{0.5}+C_{1}$$

where *k*_1_ is the intraparticle diffusion rate constant (mg/g/min), and *C*_1_ represents the boundary layer thickness effect. The values of *k*_1_ and *C*_1_ were obtained from the slope and intercept of *q_t_* versus *t*^0.5^ for the adsorption of As(III) and As(V) onto 10 wt% LDPE/AC (Figure 15A,B) and are summarized in Table 9.

The intraparticle diffusion rate constant (k_1_) ranged from 0.354 to 0.521 mg/g/min for As(III) and As(V), respectively, while the exponent *n* values ranged from 0.327 to 0.449. The intercept values C_1_ (K_diff_ on Figure 15), recorded as 2.924 for As(III) and 3.273 for As(V), describe the thickness of the boundary layer; a larger intercept indicates a greater boundary layer effect, which can enhance mass-transfer resistance and influence the overall adsorption rate. The low k_1_ values suggest that intraparticle diffusion is one of the rate-limiting steps operating alongside other mechanisms, such as surface adsorption (Pholosi, et al., 2020) [62]. High n values further reflect a favorable adsorption process. The high R^2^ values—0.802 for As(III) and 0.905 for As(V)—demonstrate that the intraparticle diffusion model describes the experimental data well, particularly for As(V). The higher R^2^ value for As(V) suggests that its adsorption process is more strongly governed by intraparticle diffusion than that of As(III).

### 3.12. Desorption and Reusability Studies

**Figure 16:**
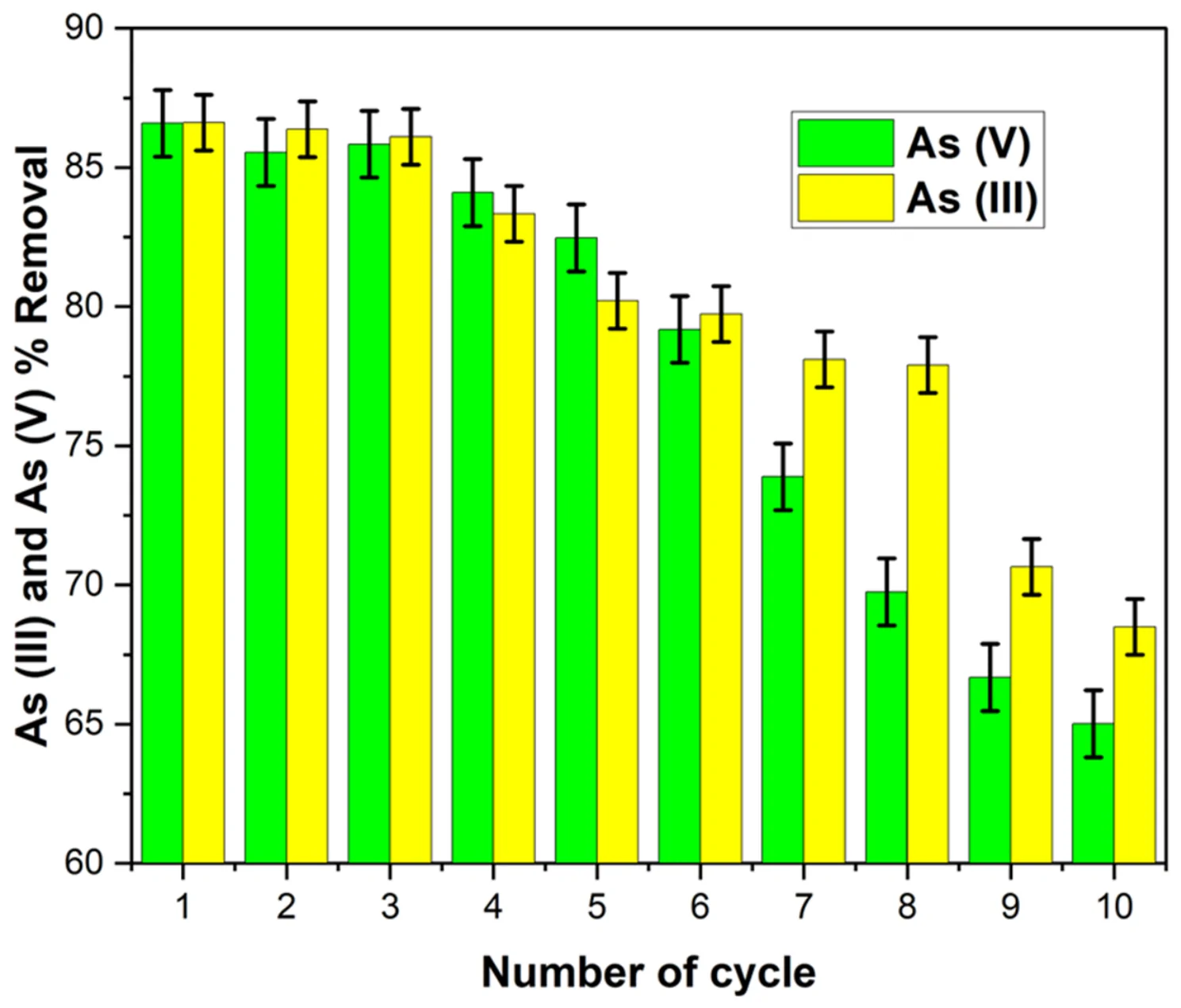
Effect of cycle time on As(III) and As(V) desorption.

Evaluating continuous adsorption–desorption cycles is essential for determining the reusability and economic viability of the adsorbent material. Figure 14 illustrates the desorption efficiency of As(III) and As(V) from 10 wt% LDPE/AC composite across ten consecutive cycles. Adsorption steps were initially conducted at 25 °C using an adsorbent dose of 0.5 g, a solution volume of 10 mL, an initial arsenic concentration of 8 mg/L, and a contact time of 30 min (at pH 4.0 ± 0.5 for As(III) and pH 8.0 ± 0.5 for As(V). Desorption experiments were subsequently carried out at 25 °C for 5 min using a 0.1 M ethylenediaminetetraacetic acid (EDTA) solution adjusted to pH 4.0. EDTA acts as a potent chelating agent that strongly interacts with the adsorbent’s active sites, disrupting the adsorbent–arsenic surface complexes and facilitating the release of arsenic species into the eluent. As shown in Figure 16, the desorption efficiency gradually decreased over ten cycles from 86 to 65.0 for As(V) and from 86.6 to 69.4 for As(III). This slight decline is attributed to incomplete desorption of strongly bound species and to the potential loss of active surface sites during repeated regeneration. Nevertheless, a retention efficiency of 83% over 4 cycles demonstrates good physical stability, whereas a decline to 69% by the fifth cycle indicates material degradation. Therefore, partial regeneration or replacement of the adsorbent is recommended after every 4 cycles to maintain optimal performance.

## 4. Conclusions

Novel low-density polyethylene/activated carbon (LDPE/AC) composite foams were successfully fabricated with AC loadings of 1, 3, 6, and 10 wt% using microcellular injection molding. Increasing the AC content enhanced both the thermal and chemical stability of the material, supported by an improved matrix microstructure. FTIR-ATR analysis confirmed the integration of key surface functional groups without altering the core structure of the parent polymer, while BET analysis demonstrated a high specific surface area of 374 m^2^/g for the 10 wt% composite foam. In batch experiments, the 10 wt% LDPE/AC composite foam demonstrated moderate adsorption removal performance for both toxic As(III) and As(V) species in aqueous solution. The adsorption equilibrium was best fitted by the Freundlich isotherm model, indicating multilayer adsorption across heterogeneous active sites. The kinetic data followed pseudo-second-order behavior, confirming that the adsorption process is chemisorption-controlled. Desorption tests using a 0.1 M EDTA solution showed that the composite foam maintained strong operational performance over 10 consecutive adsorption–desorption cycles. Overall, the study concludes that microcellular injection-molded LDPE/AC foams present a practical, scalable, and reusable solution for removing arsenic from contaminated water streams.

## Figures and Tables

**Table 1:** Process parameters for foamed LDPE/AC composites.

Process Parameter	Microcellular
Melt temperature (°C)	210
Shot size (mm)	23
Screw speed (mm/s)	25
Injection speed (mm/s)	100
Injection pressure (Bar)	1100
Mold temperature (Bar)	50
Back pressure (Bar)	100
N_2_ (%)	0.4

**Table 2:** Illustration positions of functional groups and wavenumber.

	Wave Number	Functional Group
A	2915 cm^−1^	CH_2_-Asymmetric stretch
B	2849 cm^−1^	CH_2_-Symmetric stretch
C	1472/1464 cm^−1^	CH_2_-Scissoring
D	1377 cm^−1^	CH_3_-Umbrella mode (symmetric bending)
E	730/720 cm^−1^	Split CH_2_-Rocking (crystalline splitting)

**Table 3:** BET surface area, pore volume, and average pore radius of prepared LDPE/AC.

Activated Carbon Ratio	Surface Area (m^2^/g) (sBET)	Pore Volume (cm^3^/g)	Average Pore Radius (nm)
Pure AC	1045	0.442	0.846
LDPE/AC 1 wt%	134	0.397	0.046
LDPE/AC 3 wt%	273	0.421	0.094
LDPE/AC 6 wt%	297	0.455	0.168
LDPE/AC 10 wt%	374	0.396	0.204

**Table 4:** Summary of Mechanical Properties.

Sample	Tensile Modulus (MPa)	Peak Stress (MPa)	Strain at Break (%)
Neat LDPE	2122 ± 184	8.0 ± 0.02	~73
1 wt.% AC	2167 ± 48	8.3 ± 0.01	~63
3 wt.% AC	2203 ± 59	8.1 ± 0.02	~52
6 wt.% AC	2262 ± 73	7.8 ± 0.03	~33
10 wt.% AC	2535 ± 79	7.4 ± 0.03	~31

**Table 5:** Kinetics model parameters for As(III) and As(V) adsorption on activated carbon/low-density polyethylene.

Metal	Conc.	Qe-exp	k1	k2	R^2^
Ion	(mg/L)	(mg/g)	(min^−1^)	(g/min/mg)	
As(III)	8	20.71	5.57 × 10^−4^	4 × 10^−12^	0.916
As(V)	8	24	7.35 × 10^−4^	4 × 10^−11^	0.947

**Table 6:** Comparison of different parameters from literature on activated carbon/low-density polyethylene for removing As(III) and As(V) from aqueous media.

Adsorbent	Adsorbate/Pollutant	Adsorption Capacity (mg/g)	Isotherm Model	Kinetic Model	pH	Regeneration	Reference
DMWMP *^1^	Cu(II)/Pb(II)	51.3 & 38.8	Langmuir & Freundlich	PSO	6 & 8	Not provided	[54]
Fe-Mn-Sac	As(III)	75.82	Langmuir	PSO	3	3 cycles, 0.05 M NaOH > 99%	[55]
OP/OPA *^2^	As(III)	18.60	Langmuir	PSO	7	Not provided	[56]
Limonia acidissima shell Ac	As(III)/Cu(II)	68.77 & 76.92	Langmuir	PFO & PSO	7	Not provided	[57]
IMIGAc	As(III) & As(V)	18.4 &16.0	Langmuir & Freundlich	PSO	6	Not provided	[58]
PE-g-P (Sty/MAn)	As(III)	not provided	Freundlich	PSO	7	8 cycles, 0.2 mol/L HCl	[59]
This study (LDPE/AC)	As(III) & As(V)	20.71 & 24	Freundlich	PFO & PSO	3 & 7	10 cycles 0.1 M EDTA	This study

*^1^ DMWMP: Dopamine-Modified Waste Marine Plastic; *^2^ OP/OSA: Olive Pulp and Olive Stones Ac.

**Table 7:** Classification of adsorption nature based on value.

RL Value	Type of Isotherm
RL > 1	Unfavourable
RL = 1	Linear
0 < RL < 1	Favourable
RL = 0	Irreversible

**Table 8:** Langmuir, Freundlich, and Temkin isotherm constants for the adsorption of As(III) and As(V) on activated carbon/low-density polyethylene.

	Langmuir				Freundlich			Temkin		
**Metal ions**	Qmax	RL	Kl	R2	Kf	N	R2	Bt	Kt	R2
**Unit**	(mg/g)	(-)	L m/g	(-)	L/g	(-)	(-)	J/mol	L/g	(-)
**As(III)**	20.71	0.35	0.64	0.98	5.14	0.74	0.92	1.098	19.4	0.99
**As(V)**	24	0.74	0.51	0.98	6.17	0.6	0.87	1.012	15.8	0.93

**Table 9:** McKay and Poots intraparticle diffusion parameters for the adsorption of As(III) & As(V).

Parameters	As(III)	As(V)
R^2^	0.802	0.905
K_1_ (mg·g^−1^min^−1^)	0.354	0.521
N	0.327	0.449

## Data Availability

The data presented in this study are available on request from the corresponding author.

## Abbreviations

The following abbreviations are used in this manuscript:

AC	Activated carbon
LDPE	Low-Density Polyethylene
As	Arsenic
EDTA	Ethylenediaminetetraacetic Acid
PSO	Pseudo-Second-Order
PFO	Pseudo-First-Order
